# Advanced nanomaterials for modulating Alzheimer's related amyloid aggregation

**DOI:** 10.1039/d2na00625a

**Published:** 2022-11-21

**Authors:** Xu Shao, Chaoren Yan, Chao Wang, Chaoli Wang, Yue Cao, Yang Zhou, Ping Guan, Xiaoling Hu, Wenlei Zhu, Shichao Ding

**Affiliations:** Department of Chemistry, School of Chemistry and Chemical Engineering, Northwestern Polytechnical University 127 Youyi Road Xi'an 710072 China guanping1113@nwpu.edu.cn huxl@nwpu.edu.cn; School of Medicine, Xizang Minzu University, Key Laboratory for Molecular Genetic Mechanisms and Intervention Research on High Altitude Disease of Tibet Autonomous Region Xianyang Shaanxi 712082 China; Department of Pharmaceutical Chemistry and Analysis, School of Pharmacy, Air Force Medical University 169 Changle West Road Xi'an 710032 China; Key Laboratory for Organic Electronics & Information Displays (KLOEID), Institute of Advanced Materials (IAM), Nanjing University of Posts & Telecommunications (NJUPT) Nanjing 210046 China; School of the Environment, School of Chemistry and Chemical Engineering, State Key Laboratory of Analytical Chemistry for Life Science, State Key Laboratory of Pollution Control & Resource Reuse, Nanjing University Nanjing 210023 P. R. China wenleizhu@nju.edu.cn; School of Mechanical and Materials Engineering, Washington State University Pullman WA 99164 USA shichao.ding@wsu.edu

## Abstract

Alzheimer's disease (AD) is a common neurodegenerative disease that brings about enormous economic pressure to families and society. Inhibiting abnormal aggregation of Aβ and accelerating the dissociation of aggregates is treated as an effective method to prevent and treat AD. Recently, nanomaterials have been applied in AD treatment due to their excellent physicochemical properties and drug activity. As a drug delivery platform or inhibitor, various excellent nanomaterials have exhibited potential in inhibiting Aβ fibrillation, disaggregating, and clearing mature amyloid plaques by enhancing the performance of drugs. This review comprehensively summarizes the advantages and disadvantages of nanomaterials in modulating amyloid aggregation and AD treatment. The design of various functional nanomaterials is discussed, and the strategies for improved properties toward AD treatment are analyzed. Finally, the challenges faced by nanomaterials with different dimensions in AD-related amyloid aggregate modulation are expounded, and the prospects of nanomaterials are proposed.

## Abstract 2

Based on the dimensional scale, this review summaries the use of nanomaterials in the modulation of amyloid aggregation. The effects include intermolecular interaction, chelation, photothermal effect, photocatalytic oxidation, and drug delivery.

## Graphical abstract

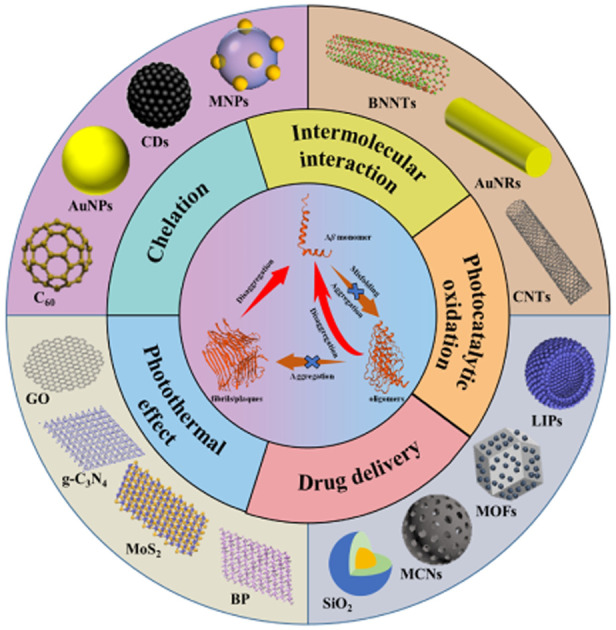

## 1 Introduction

Protein misfolding can form abnormal amyloid aggregates, further leading to amyloid extracellular deposition.^1^ These amyloid deposits are widely believed to be closely related to various neurodegenerative diseases and are even considered to be the culprit.^2^ Among them, Alzheimer's disease (AD) is the most common form of neurodegenerative disease, and according to the “2021 World Alzheimer's Disease Report”, more than 55 million people worldwide have dementia. This number gets even more staggering as it grows daily and is expected to reach 78 million by 2030.^3^ Although the pathogenesis of AD has not been clearly confirmed, the amyloid plaque hypothesis has been the most widely accepted until now.^4^ As the most important component of amyloid aggregates, Aβ is derived from the sequential proteolytic cleavage of β-amyloid precursor protein (APP) by β- and γ-secretase *in vivo*.^5^ In addition, Aβ is a hydrophobic peptide with a molecular weight of 4 kDa and consists of 39–42 amino acid residues.^5,6^ The Aβ monomer undergoes secondary structural transitions and misfolds in physiological environments.^7^ This misfolding Aβ can rapidly self-assemble with surrounding Aβ and form oligomers through hydrophobic interactions. Meanwhile, the oligomers can then decrease through the conversion of non-fibrillar to fibrillar oligomers, elongating fibrillar oligomers and finally forming mature amyloid fibrils.^8^ The process of Aβ aggregation can trigger the production of intra- and extra-cellular reactive oxygen species (ROS), which can lead to oxidation and cellular damage.^9^ In addition, neurotoxicity was induced by Aβ oligomers and fibrils through binding to the plasma membrane, resulting in metabolic dysfunction and neuronal cell death.^10^ Therefore, the inhibition of Aβ fibrillation, the disintegration of mature Aβ aggregates, and the promotion of the clearance of Aβ to maintain the balance of the metabolism and catabolism of Aβ appear to be quite significant for the prevention and treatment of AD. Recently, numerous efforts have been made to inhibit Aβ aggregation by blocking fibril formation and reducing the number of fibrils to halt the extent of AD pathology.^10–12^ Among them, nanomaterials have great advantages in influencing amyloid fibril nucleation, disintegrating matured amyloid fibrils, and targeting amyloid plaques *via* crossing the blood–brain barrier (BBB).^10,13–16^ At the same time, nanomaterials have an ability to respond to light, sound, heat, electricity, and magnetism because of the physical properties of some nanomaterials, and they have also been gradually developed and applied in the research of neurodegenerative diseases.^10,17–21^

**Fig. 1:**
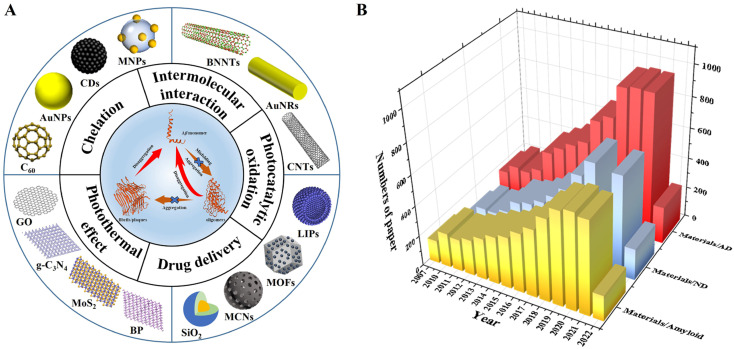
(A) Schematic illustration of nanomaterials with different functions and dimensions for modulating Aβ aggregation. (B) The number of published papers per year on the application of nanomaterials in amyloid, neurodegenerative disease (ND), and Alzheimer's disease (AD). Data are collected from the Web of Science on June 26, 2022, by advanced search with “Topics = (Materials and Amyloid; Materials and Neurodegenerative Disease; Materials and Alzheimer's disease; Language: (English)”.

Nanomaterials can be classified into one-dimensional, two-dimensional, zero-dimensional, and other nanomaterials according to their dimensions.^22^ One-dimensional nanomaterials exhibit a high degree of anisotropy, possessing excellent properties such as plasmon resonance, optical properties and anti-oxidation.^23^ Two-dimensional nanomaterials have excellent physical and chemical properties, can bind peptides through non-covalent forces, have good biocompatibility, and have good photothermal conversion and photocatalytic capabilities.^24–26^ The large specific surface area of zero-dimensional nanomaterials makes them have unique physical and chemical properties.^27^ Besides, some composite nanomaterials prepared from other nanocarriers, such as metal–organic frameworks, polyoxometalates, and silica, have multiple synergistic effects.^28–30^ Based on the three-dimensional scale of nanomaterials, this review deeply analyzed the advantages/disadvantages of nanomaterials in modulating amyloid aggregation. The modulation roles of nanomaterials in AD treatment mainly include intermolecular interaction, chelation, photothermal effects, photocatalytic oxidation, and drug delivery (Fig. 1A). As shown in Fig. 1B, we exhibited a number of research articles published each year on the application of nanomaterials in amyloid, neurodegenerative disease (ND), and Alzheimer's disease (AD). This exponential growth of research in the related field indicates that nanomaterials for modulating Alzheimer's related amyloid aggregation are not only an emerging research topic, but also possess huge application potential.

## 2 One-dimensional nanomaterials

One-dimensional (1D) nanomaterials, including nanorods, nanotubes, nanoribbons, nanowires, and nanofibers, have been applied as drug carrier or synergistic drug materials.^31,32^ Due to their unique chemical structures, good biocompatibility, high specific surface area, and other related physicochemical properties, 1D nanomaterials were widely applied to the biological field.^33^ In recent years, some research showed that 1D nanomaterials with special structures, such as radial size seamless carbon tubes, can interact with amyloid protein and reduce the aggregation of amyloid protein.^34^

### 2.1 Carbon nanotubes

**Fig. 2:**
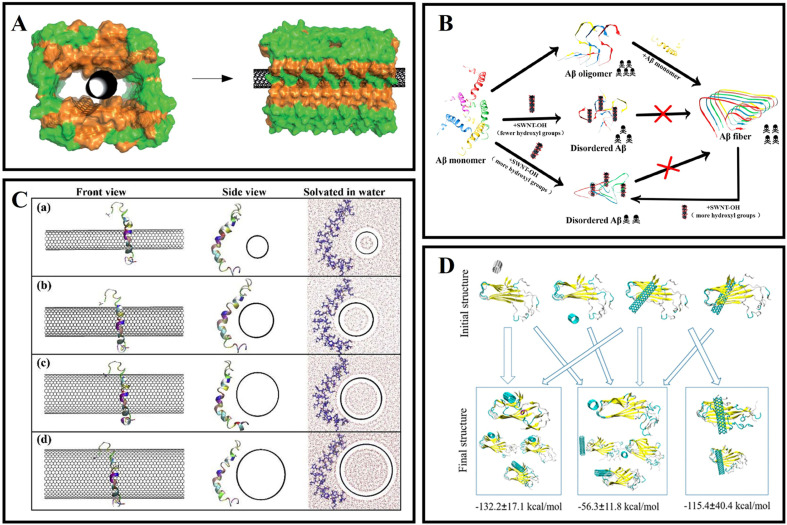
(A) Illustration of SWNTs located in the hollow core of Aβ fibrils.^45^ (B) Hydroxylated SWNTs inhibit Aβ_42_ fibrillogenic and disaggregate mature fibrils.^48^ (C) Initial configurations of the Aβ_42_ peptide with SWCNT chiralities of (a) (10, 10), (b) (15, 15), (c) (20, 20) and (d) (25, 25).^49^ (D) Disaggregation process of Aβ_42_ fibrils-SWCNTs in 200 ns.^50^

Carbon nanotubes with a special structure fabricated from graphene sheets are one-dimensional quantum materials.^35^ It is mainly composed of several to dozens of layers of coaxial circular tubes of carbon atoms arranged in a hexagonal shape.^36^ A fixed distance of about 0.34 nm is maintained between layers, and the diameter of nanotubes is generally 2–20 nm.^37^ According to the different orientations of the hexagon along the axial direction, it can be divided into zigzag, armchair and spiral.^38^ Single-walled carbon nanotubes (SWCNTs) have been applied in various biological systems because of their good biocompatibility, unique chemical structure, high specific surface area and strong optical absorbance in the near-infrared (NIR) region.^39^ As unique one-dimensional nanomaterials, SWCNTs have also been explored as novel delivery vehicles for drugs, proteins, and so on.^40,41^ Due to the strong optical absorbance of SWCNTs in the NIR region, SWCNTs could destroy the structure of cells by local thermal during NIR laser irradiation.^42^ As a nanocarrier, SWCNTs were used to deliver oligonucleotides into living cells, and oligos were translocated into cell nuclei upon endosomal rupture triggered by NIR laser pulses.^43,44^ It can be seen that the transporting capabilities of SWCNTs combined with chemical modification and their intrinsic optical properties can lead to new classes of novel nanomedicine for drug delivery and therapy. To the best of our knowledge, SWCNTs have also been developed for inhibiting amyloid fibrillation, disintegration of amyloid fibrils, and promoting the clearance of amyloid plaques. Luo *et al.*^45^ firstly studied the pH-dependent molecular interactions between SWCNTs and Aβ peptides by a variety of spectroscopy and atomic force microscopy techniques. They found that the secondary structural transition of Aβ peptides from a random coil to a β-sheet structure could be significantly affected by SWCNTs, and SWCNTs could inhibit the nucleation/elongation phase of Aβ peptide fibrillation by adsorbing Aβ peptides with a β-sheet structure (Fig. 2A). Their research also indicated that Aβ peptides might reduce the toxicity of SWCNTs by the reduction of the hydrophobic surface of SWCNTs. Wei's group^46^ showed that SWCNTs could inhibit the formation of β-sheet-rich oligomers in the central hydrophobic core fragment of Aβ (Aβ_16–22_). However, a potential problem with SWCNTs is their poor solubility in water and few functional groups, which will cause a huge hindrance to the inhibition of Aβ fibrillation and other biological applications. Therefore, Xie *et al.*^47^ fabricated a type of hydroxylated SWCNTs by modifying with 30 hydroxyl groups. Then they further investigated the influence of hydroxylated SWCNTs on the aggregation of Aβ_16–22_ peptides using all-atom explicit-water replica exchange molecular dynamics simulations. The results showed that the β-sheet formation, shift in the conformations and disordered aggregation of Aβ_16–22_ peptides can be significantly inhibited through hydroxylated SWCNTs, which mainly depend on the strong electrostatic, hydrophobic, and aromatic stacking interactions with the residue of Aβ_16–22_. In addition, Liu *et al.*^48^ also researched the ability of hydroxylated SWCNTs for inhibiting Aβ aggregation, disaggregating Aβ fibrils, and protecting Aβ-induced cytotoxicity. The authors found that SWCNT-OH could inhibit Aβ fibrillation and disaggregate mature fibrils in a dose-dependent manner (Fig. 2B). Moreover, the related experience showed that the ratio of hydroxyl groups in SWCNT-OH played an important role in inhibiting Aβ fibrillation. In detail, with the increase the ratio of hydroxyl groups, the inhibitory capacity of SWCNT-OH was greatly improved. Molecular dynamics (MD) simulations further revealed that the interactions between SWCNT-OH and the Aβ_11–42_ pentamer were found to be dominated by van der Waals interactions. In addition, the inter- and intra-peptide interactions of Aβ fibrillation were significantly weakened by hydrophobic interactions and π–π stacking of Aβ and SWCNT-OH, and SWCNT-OH mainly interact with the six residues of Aβ_11–42_ (H13, H14, Q15, V36, G37, and G38). In our group, the structure of the Aβ_42_ monomer affected by tuning the curvature of carbon nanotubes was deeply studied using MD simulations.^49^ The related research indicated that Aβ_42_ peptides had an extended structure and a larger number of contacts with the surface of C25. When the curvatures of the carbon nanotubes (CNTs) were high, the peptide wrapped around the CNTs and had less contact with the surfaces (Fig. 2C). Moreover, the CNTs with lower curvatures and the peptides had stronger interactions and induced the collapse of the initial secondary structures of the peptides. With decreasing curvatures, the peptides were arranged diagonally along the nanotube, and the percentages of α-helical structures were reduced. This research indicated that the structural stability, including the nucleation and self-assembly behavior of Aβ_42_ peptides on SWCNT surfaces, is dependent on the surface curvatures. The disaggregation mechanism of SWCNTs for mature Aβ fibrils was also investigated. For instance, Lin *et al.*^50^ explored the interplay between SWCNTs and Aβ fibrils by atomic force microscopy, ThT fluorescence, infrared spectroscopy, and MD simulations at the single SWCNT level. The results demonstrated that SWCNTs could partially destroy the mature Aβ fibrils and form Aβ-surrounded-SWCNT conjugates and cut down the β-sheet structures. Besides, MD simulation confirmed that the disaggregation ability was dependent on the binding sites of Aβ fibrils (Fig. 2D).

Compared to SWCNTs, multiwalled carbon nanotubes (MWCNTs) possess obvious advantages, such as lower product cost, excellent chemical stability and drug adsorption potential.^51^ Lohan *et al.*^52^ designed a system of berberine (BRB)-loaded MWCNTs with polysorbate and phospholipid coating. BRB was known to possess neuroprotective actions. Polysorbates and phospholipids have been reported to improve the imaging and targeting utility of CNTs. The results showed that the phospholipid-coated and the polysorbate-coated MWCNTs exhibited remarkable recovery in the memory performance.

### 2.2 Gold nanorods

Gold nanorods are rod-shaped gold nanoparticles with a size ranging from a few nanometers to hundreds of nanometers.^53^ Gold is a precious metal material with very stable chemical properties. Gold nanoparticles inherit these properties of bulk materials, so they are relatively stable and have very rich physicochemical properties.^54^ The surface plasmon resonance wavelength of gold nanorods can be changed with the aspect ratio, continuously adjustable from visible (550 nm) to near-infrared (1550 nm), and an extremely high surface electric field strength enhancement effect.^55,56^ Gold nanorods have extremely high optical absorption, scattering cross-sections, and photothermal conversion efficiency that is continuously adjustable from 50% to 100%.^57,58^ Therefore, Au nanorods (AuNRs) exhibit strong localized surface plasmon resonance (LSPR) in the near-infrared spectrum and have good performance in photothermal (PTT) therapy.^59,60^

**Fig. 3:**
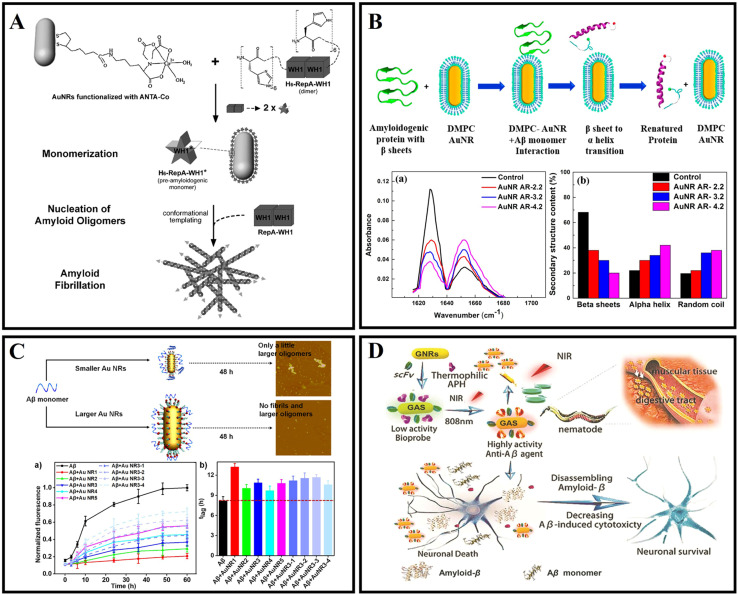
(A) Illustration of the nucleation of RepA-WH1 amyloidogenesis by prionoid-functionalized AuNRs.^61^ (B) Interaction between AuNRs and Aβ protein monomer and conversion of the β-sheet to an α-helix secondary structure.^63^ (C) CTAB-stabilized AuNRs with different sizes inhibiting Aβ peptide aggregation.^64^ (D) The GAS with NIR absorption is used for AD diagnosis and treatment.^65^

Gold nanorods as potential therapy nanomaterials have been utilized to modulate amyloid aggregation. AuNRs were functionalized with a metal-chelating group amide-nitrilotriacetic-Co^II^ (ANTACo) to immobilize soluble RepA-WH1 selectively (Fig. 3A). In the presence of catalytic concentrations of anisotropic nanoparticles, H6-RepA-WH1 undergoes stable amyloid oligomerization.^61^ Then, such oligomers promote the growth of amyloid fibers of untagged RepA-WH1. Prionoid-functionalized AuNRs as nucleating agents for controlled protein amyloidosis *in vitro*. AuNR-mediated amyloid nucleation is based on a conformational change from the dimer protein precursor to the immobilized pre-amyloidogenic monomer at the nanoparticle surface, which effectively promotes the oligomerization and fibrillation of amyloid. Lin *et al.*^62^ introduced a novel method where AuNRs combined with Aβ fibrils can be efficiently destroyed under fs-laser irradiation without increasing the cytotoxicity. The fs-laser could trigger the nanoexplosion of AuNRs by LSPR and bring the Aβ fibrils into non-β-sheet structure components. Sudhakar *et al.*^63^ fabricated AuNRs and utilized them to inhibit the aggregation of Aβ by a NIR laser. Meanwhile, the shape-dependent plasmonic properties of AuNRs are exploited to facilitate faster disaggregation of mature Aβ fibrils. In addition, a related study found that 1,2-dimyristoyl-*sn-glycero*-3-phosphocholine (DMPC) stabilized AuNRs can inhibit the formation of fibrils due to selective binding to the positively charged amyloidogenic sequence of Aβ protein (Fig. 3B). This research exhibited a dual effect: inhibition of Aβ fibrillation and NIR laser facilitated the dissolution of mature Aβ fibrils. However, the role of heat generation by AuNRs, which promoted the disaggregation of fibrils, had not been explained from a molecular perspective.^63^ Then Liu *et al.*^64^ prepared CTAB-stabilized AuNRs with different sizes (CTAB as cetyltrimethylammonium bromide), and the effect of diameters and lengths of AuNRs on Aβ fibrillation was in-depth studied. A related fluorescence experiment indicated that in the presence of CTAB-stabilized AuNRs with different sizes, the formation of larger oligomers and fibrils was inhibited, and the inhibition efficiency decreased with the decrease of diameters of AuNRs (Fig. 3C). For the AuNRs with the same diameter, the inhibition efficiency decreased with the length of Au NRs. A CD experiment indicated that AuNRs with larger sizes inhibited the formation of a β-sheet structure to some extent. In summary, CTAB-stabilized AuNRs inhibited the kinetic process of Aβ fibrillation, and the inhibition efficiency of larger AuNRs was better. Meanwhile, the sizes of AuNRs played a key role in modulating the kinetic aggregation process of Aβ fibrillation. This work found that the rate constant had a positive relationship with the diameters or lengths of CTAB-stabilized AuNRs. Interestingly, Liu *et al.*^65^ studied the NIR absorption properties of AuNRs loaded with a single chain variable fragment and thermophilic acylpeptide hydrolase as a smart theranostic complex GAS, which possesses both rapid detection of Aβ aggregates and NIR photothermal treatment that effectively disaggregates Aβ aggregates and reduces Aβ-mediated toxicity (Fig. 3D). Morales-Zavala *et al.*^66^ synthetized a polyethylene glycol stabled and dual-peptide modified gold nanorod complex. A related study determined that the nanoconjugate does not affect neuronal viability. The nanoconjugate could penetrate the cells and decrease the Aβ peptide aggregation *in vitro*. Subsequently, Morales-Zavala *et al.*^67^ also developed a neurotheranostic platform based on AuNRs, which works as a therapeutic peptide delivery system. As a diagnostic tool, the platform could be detected *in vivo* through microcomputed tomography (micro-CT). Ang2 and D1 peptide modified AuNRs induced the diminution of both the amyloid load and inflammatory markers in the brain of the AD model. The differences in GNRs-D1/Ang2 between wild type (WT) and AD mice were observed *in vivo*. The two peptide modified AuNRs can improve the delivery and retention of this platform in the brain and reinforce the therapeutic benefits associated with the β-sheet breaker ability of the D1 peptide.

## 3 Two-dimensional nanomaterials

Two-dimensional (2D) nanomaterials refer to nanomaterials that have only one dimension on the nanometer scale.^68^ Because of their huge specific surface area and special surface structure, 2D nanomaterials can adsorb and interact with various molecules such as drugs, nucleic acids, peptides, and proteins.^69^ Two-dimensional nanomaterials also have the ability to penetrate biological barriers.^70^ Therefore, as a drug carrier, 2D nanomaterials can load numerous drugs and cross various biological barriers.^71,72^ Meanwhile, 2D nanomaterials can also absorb and immobilize amyloid protein by interacting with interfaces.^73^ Some 2D nanomaterials possess light-responsive properties and have great potential in photothermal and photodynamic therapy.^69,74^ Two-dimensional nanomaterials have good peroxidase-like properties and can alleviate oxidative stress.^75^ Based on the advanced properties, 2D nanomaterials have been attractive in AD diagnosis and treatment.^76^ 2D nanomaterials have been used in AD research, mainly including graphene nanosheets, carbon nitride nanosheets, black phosphorus nanosheets, and transition metal dichalcogenide nanosheets. Besides, some studies have shown that 2D MOFs, MXenes, hexagonal boron nitride and so on also have applications in AD diagnosis and treatment.

### 3.1 Graphene

Graphene or graphene oxide (GO), one of the two-dimensional nanomaterials, consists of mono-layer carbon atoms with conjugated π–π.^77^ Due to the excellent electrical conductivity, ultra-high specific surface area, high mechanical strength, good biocompatibility, and photothermal conversion characteristics, graphene has been widely used in biomedical fields such as bioimaging, biosensing, and drug delivery.^78–80^

**Fig. 4:**
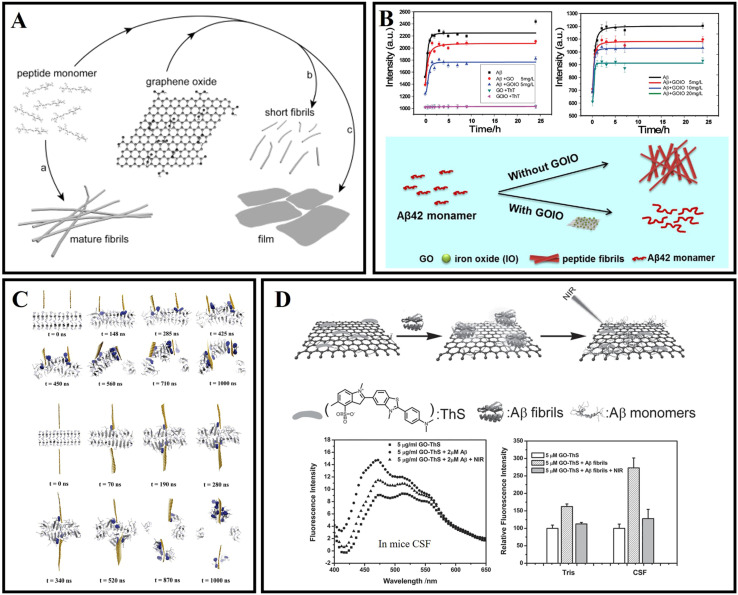
(A) The surface of graphene-oxide sheets redirects the amyloid–peptide assembly process.^82^ (B) Kinetics of Aβ_42_ fibrillation and illustration of modulation of Aβ_42_ aggregation by using GOIO.^84^ (C) Graphene nanosheet penetration and Aβ peptide extraction. Featuring two graphene sheets attacking a pre-formed Aβ amyloid fibril from the same side, and the two graphene sheets attacking from both sides.^85^ (D) GO–ThS effectively dissolve the amyloid deposits of Aβ_40_ upon NIR laser irradiation.^91^

Mahmoudi *et al.*^81^ indicated that GO and protein-coated GO can delay the Aβ fibrillization process *via* adsorption of amyloid monomers. Then Li *et al.*^82^ further confirmed that the binding between the peptide monomer and the surface of the GO sheets can redirect the assembly pathway of Aβ (Fig. 4A). Wang *et al.*^83^ examined the size effect of GO on modulating amyloid peptide assembly and found that GO with a large size has a relatively stronger modulation effect for the aggregation of Aβ_33–42_. The advantages of graphene nanocomposites are even more obvious. As shown in Fig. 4B, Ahmad *et al.*^84^ successfully fabricated nanocomposites of iron oxide and graphene oxide (GOIO) using solvothermal methods. Due to the high surface area of GOIO, GOIO can effectively interact with Aβ_42_, inhibit the formation of mature fibrils from Aβ_42_ monomers and maintain the secondary structure of Aβ_42_ into a random coil or α-helix-rich structure. Many researchers have worked to investigate the mechanism of action of graphene bias with Aβ. The penetration and extraction of graphene were identified as two main mechanisms for scavenging fibrils (Fig. 4C).^85^ This is because of the strong interaction between graphene and amyloid fibrils through π–π stacking and hydrophobic interaction due to the special sp^2^ structure of graphene. Graphene nanosheets can extract single peptide molecules from mature amyloid fibrils into their surface, and the absorption interaction is further enhanced by π–π stacking because of the aromatic residues of Aβ and the sp^2^ structure of graphene. Chen *et al.*^86^ investigated the oligomerization of Aβ_33–42_ by performing replica exchange MD simulations on Aβ_33–42_ peptide chains in the absence and presence of two different sizes of GO, and found that GO inhibited Aβ_33–42_ oligomerization by making Aβ_33–42_ peptides separate from each other. Jin *et al.*^87^ revealed the mechanism of GO nanosheets in inhibiting Aβ_42_ aggregation through MD simulations, and found that GO mostly suppressed the β-sheet formation of Aβ_42_ by weakening inter-peptide interactions mostly *via* the salt bridge, hydrogen bonding and cation–π interactions with charged residues D1, E3, R5, D7, E11, K16, E22, K28 and A42. The π–π and hydrophobic interactions between GO and Aβ_42_ also play a key role in the inhibition of Aβ aggregation. Meanwhile, Yin *et al.*^88^ indicated that the adsorption capacity with Aβ of graphene's surface varies significantly depending on its curvature. The negative curved surface is more likely to adsorb Aβ than the positive curved surface. These findings showed that the shape of the nanoparticle is important in determining its interaction with the peptide. He *et al.*^89^ investigated the thermodynamics and kinetics of fibril elongation on GO surfaces with different oxidative degrees. This study revealed that the behaviors of GO in fibril elongation depend on the balance between the promoting effect by templating the incoming of monomers and the retarding effect by capturing the monomer during docking and locking phases through hydrogen bonding. Subsequently, Li *et al.*^90^ also further demonstrated that GO could clear amyloids by inducing microglia and neuron autophagy. Photothermal therapy can be used to dissolve mature Aβ fibrils. As shown in Fig. 4D, Qu's group firstly reported the photothermal treatment for AD using graphene nanosheets. Thioflavin S (ThS) which can specifically bind to Aβ fibers was covalently linked to the surface of GO. The prepared GO–ThS nanocomposites have a uniform diameter of 100 to 200 nm, and the thickness of GO–ThS nanocomposites is about 1.5 nm. The related research showed that GO–ThS can cross the BBB, selectively interact with Aβ_40_ fibrils, and disaggregate Aβ_40_ fibrils under near-infrared (NIR) laser irradiation. Moreover, the decomposition of Aβ_40_ fibrils can be monitored by the fluorescence changes of ThS in real time.^91^ Xia and Maciel *et al.*^92,93^ have reported a potential drug carrier for loading drugs using GO through non-covalent interactions. Wang *et al.*^94^ prepared a novel nanocomposite GO@Dau from GO and dauricine (Dau), and the benzene ring on Dau can be adsorbed by GO by forming a non-covalent bond. GO@Dau will both have anti-inflammatory and anti-oxidative stress capabilities and inhibit Aβ misfolding. This study further found that GO@Dau can effectively enrich in the brain after intranasal administration and GO@Dau can be internalized into the olfactory bulb by endocytosis or pinocytosis of olfactory neurons, and then released and distributed into the brain. More interestingly, researchers found that GO@Dau could increase superoxide dismutase levels, decrease reactive oxygen species and malondialdehyde levels *in vitro*, and attenuate cognitive memory deficits and glial cell activation for AD mice.

Overall, graphene nanosheets and their nanocomposites have been reported for use in AD therapy. However, the specific-targeted issue and drug delivery modalities of graphene still need to be elucidated. Especially, the BBB penetration of graphene is needed to be deeply researched. Through functionalization and size or shape adjustment for nanomaterials, utilizing paracellular pathway, transcellular lipophilic pathway, transport proteins, receptor-mediated transcytosis, and adsorptive-mediated transcytosis could achieve penetration of the BBB.^95,96^ Although many investigators have studied and summarized the biodistribution characteristics, *in vivo* clearance, toxicity, and interactions with biological systems of GO, there is still much to be unveiled that would allow safe and effective therapy.^97,98^

### 3.2 g-C_3_N_4_

Graphitic carbon nitride (g-C_3_N_4_) is the most stable allotrope of carbon nitride under ambient conditions.^99^ g-C_3_N_4_ has thermodynamic stability, good biocompatibility, low toxicity, and unique photocatalytic properties.^100–102^ It has received extensive attention in biological applications in recent years.^103^

**Fig. 5:**
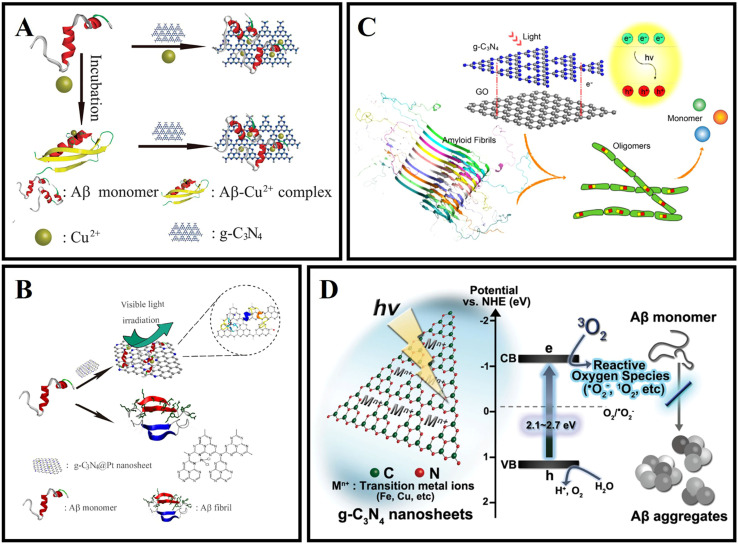
(A) The ultrathin g-C_3_N_4_ nanosheets can effectively inhibit Cu^2+^ induced Aβ aggregation and disaggregate the preformed Aβ–Cu^2+^ aggregates.^104^ (B) g-C_3_N_4_@Pt was used for AD treatment.^105^ (C) The disaggregation of Aβ aggregates by GO/g-C_3_N_4_ under light irradiation.^106^ (D) Highly reactive ROS trigger peptide oxidation that suppresses further fibril formation of Aβ.^108^

In 2016, Li *et al.* firstly used g-C_3_N_4_ as an Aβ inhibitor for AD treatment.^104^ As shown in Fig. 5A, g-C_3_N_4_ nanosheets could effectively inhibit the formation of Aβ aggregates, separate the preformed Aβ–Cu^2+^ aggregates, and reduce the intracellular reactive oxygen species (ROS) levels. Then, Li *et al.*^105^ combined the advantages of g-C_3_N_4_ nanosheets with some metal complexes to fabricate platinum(ii)-coordinated g-C_3_N_4_ nanosheets (g-C_3_N_4_@Pt), and g-C_3_N_4_@Pt was able to inhibit Aβ fibrillation. As shown in Fig. 5B, g-C_3_N_4_@Pt could effectively inhibit the aggregation of Aβ through non-covalent interaction and photooxidation. As shown in Fig. 5C, Wang *et al.*^106^ prepared a nanocomposite which is named GO/g-C_3_N_4_ by the sonochemical method. Under UV light irradiation, GO/g-C_3_N_4_ could disaggregate mature Aβ fibrils. GO could act as an Aβ collector by adsorption interaction and g-C_3_N_4_ could serve as a cleaner by photodegradation. Notably, the photodegradation efficiency of the composite could be kept high because the heterojunction between GO and g-C_3_N_4_ helps to separate the photoexcited electron–hole pairs. In 2020, Wang *et al.*^107^ reported a kind of novel gold nanoparticle modified g-C_3_N_4_ (Au/g-C_3_N_4_), which can effectively degrade preformed amyloid aggregates, and the photodegradation of amyloid aggregates mainly depends on the generation of oxygen radicals, especially hydroxyl radicals. As shown in Fig. 5D, Chung *et al.*^108^ verified that g-C_3_N_4_ can effectively inhibit the aggregation of Aβ under light illumination. Under visible light irradiation, g-C_3_N_4_ nanosheets could generate ROS through photo-induced electron transfer, and oxidize Aβ protein, preventing Aβ misfolding and fibrillation. The inhibition efficiency of g-C_3_N_4_ for Aβ aggregation will be increased with the concentration and absorbance intensity of g-C_3_N_4_ under LED irradiation. Doping metal ions, such as iron, can help g-C_3_N_4_ nanosheets accelerate the charge transfer activity, resulting in high ROS generation for inhibiting Aβ aggregation.^109^

g-C_3_N_4_ has some inherent disadvantages, such as poor water solubility, relatively large particle size, and lack of absorption above 460 nm, but its reliable biocompatibility at certain doses proves its potential for biological applications.^110–112^ For g-C_3_N_4_ applications in living organisms, issues such as autofluorescence, optical therapeutic efficiency, and *in vivo* clearance rates still need to be addressed.^103^

### 3.3 Black phosphorus

Black phosphorus (BP) nanosheets, a novel two-dimensional layered semiconductor nanomaterial, have attracted extensive attention due to their good optical, thermal properties, photocatalytic properties, and biological compatibility.^113,114^ BP can be degraded into non-toxic phosphate and phosphite anions under physiological conditions.^115^ BP nanosheets can efficiently and selectively capture Cu^2+^ to protect neuronal cells from Cu^2+^-induced neurotoxicity.^116^ Moreover, due to the photothermal transition efficiency, BP nanosheets can cross the BBB by relying on NIR laser irradiation.^117^

**Fig. 6:**
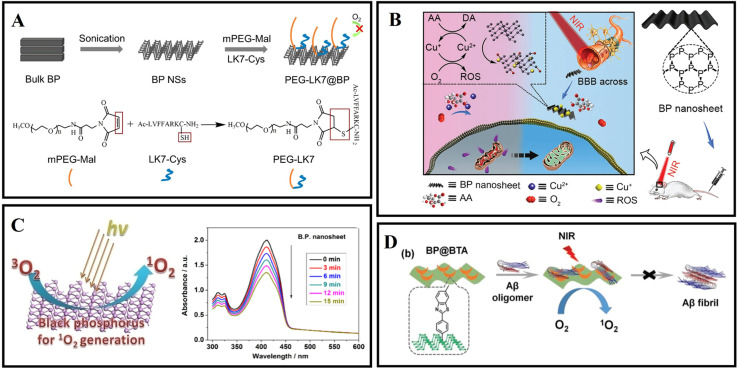
(A) The preparation of PEG-LK7@BP and the reaction of mPEG-Mal with LK7-Cys during PEG-LK7@BP formation.^119^ (B) BP nanosheets as a BBB penetrable nanocaptor to reduce oxidative stress production through capturing Cu ions.^121^ (C) Ultrathin BP nanosheets for efficient singlet oxygen generation.^125^ (D) BP@BTA produced ^1^O_2_ under NIR to inhibit Aβ aggregation.^126^

In 2019, Lim *et al.*^118^ synthesized two kinds of typical BP nanomaterials with different sizes, titanium ligand-modified BP nanosheets (TiL_4_@BPNSs) and titanium ligand-modified BP quantum dots (TiL_4_@BPQDs). The results showed that TiL_4_@BPNSs and TiL_4_@BPQDs inhibited Aβ_40_ aggregate by adsorbing Aβ_40_ monomers. Then, Yang *et al.*^119^ designed a PEG-stabilized BP nano-system PEG-LK7@BP, which can effectively inhibit the formation of Aβ_42_ fibrils (Fig. 6A). In addition, as a peptide inhibitor, LK7 was coupled to the BP surface *via* electrostatic and p–π interactions. PEG was used to enhance the stability of BP. PEG-LK7@BP inhibited Aβ_42_ fibrillation in a dose-dependent manner. Importantly, PEG-LK7@BP has no cytotoxicity to normal cells and can effectively alleviate the cytotoxicity induced by Aβ. The inhibition ability of PEG-LK7@BP can be attributed to multiple effects: (1) PEG-LK7@BP can bind with Aβ through electrostatic and hydrophobic interactions. (2) LK7 can enhance the targeted properties of PEG-LK7@BP for Aβ amyloid. (3) PEG enhanced the stability and dispersibility of the nanomaterials. Cu^2+^ can catalyze the production of ROS and cause neuronal apoptosis.^120^ Therefore, it is needed to design novel nanomaterials for not only capturing excess metals but also crossing the BBB. As shown in Fig. 6B, Chen *et al.*^121^ demonstrated that BP nanosheets can efficiently and selectively chelate Cu^2+^ to inhibit neurotoxicity induced by Cu^2+^. Importantly, under the irradiation of a NIR laser, the BBB permeability of BP nanosheets is significantly improved due to the photothermal effect.

Due to the properties of precise treatment and fewer side effects for various diseases, photodynamic therapy (PDT) has attracted extensive attention in the biomedical field.^122,123^ However, some photosensitizers suffer from low catalytic efficiency, a short absorption wavelength, poor biocompatibility, and non-degradability in living tissues.^124^ In 2015, Wang *et al.*^125^ first demonstrated that exfoliated BP nanosheets are effective photosensitizers for generating ^1^O_2_, and the quantum yield is about 0.91 (Fig. 6C). These excellent properties make BP nanosheets photocatalysis nanomaterials in PDT therapy. As shown in Fig. 6D, Qu's group designed a near-infrared responsive nanomaterial based on BP nanosheets.^126^ The authors also utilized BTA (one of the thioflavin-T derivatives) to modify black phosphorus, aiming to recognize Aβ and enhance BP stability. BP@BTA could generate ^1^O_2_ efficiently and the inhibition efficiency of Aβ fibrillation was effectively heightened.

Compared with other 2D materials, BP exhibits a tunable energy bandgap from about 0.3 eV (bulk) to 2.0 eV (monolayer), allowing broad absorption across the entire ultraviolet and infrared regions.^127,128^ Moreover, the degradable character of BP from element to nontoxic and biocompatible phosphorus oxides is endowed with good biocompatibility *in vivo*.^129^

### 3.4 Transition metal dichalcogenides

Different from carbon or phosphorus-based two-dimensional (2D) nanomaterials, transition metal dichalcogenide nanosheets have become alternative candidates, such as MoS_2_ and WS_2_. MoS_2_ and WS_2_ are sandwich structures composed of hexagonal metal atoms sandwiched between two layers of chalcogens.^130^ Transition metal dichalcogenide nanosheets were shown to address biological and medical fields due to their novel nanoscale structures, rich physics, and high mobility.^131–133^ The basal plane of transition metal disulfide nanosheets can adsorb or conjugate various aromatic hydrocarbons (such as pyridine and purine) and other compounds.^134^ In recent years, transition metal dichalcogenide nanosheets have been reported for drug delivery and tissue ablation.^135^

**Fig. 7:**
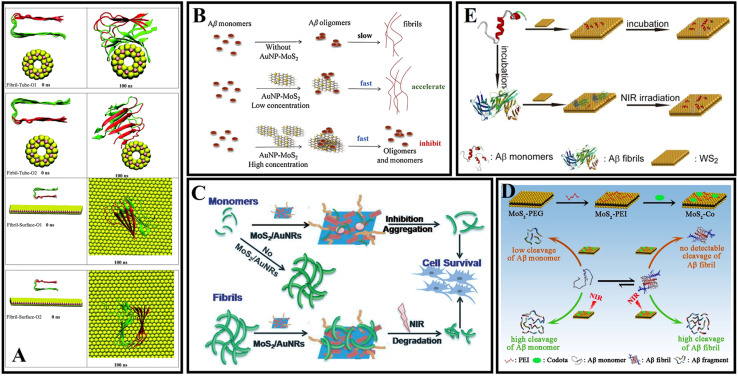
(A) Initial and final snapshots of the interaction between amyloid fibrils and MoS_2_ nanomaterials.^138^ (B) Concentration-dependent mechanism of AuNP-MoS_2_ nanocomposites in Aβ_40_ aggregation.^139^ (C) MoS_2_/AuNR nanocomposites with high NIR absorption were used for inhibiting β-amyloid aggregation.^140^ (D) MoS_2_-Co improved the hydrolytic activity toward Aβ monomers and enhanced the hydrolytic capacity toward Aβ fibrils in the presence of a NIR laser.^141^ (E) WS_2_ nanosheets with high NIR absorbance are used for AD treatment.^142^

In 2013, Chou *et al.*^136^ prepared MoS_2_ by a chemical exfoliation method and obtained a two-dimensional amphiphilic compound with good colloidal stability in aqueous media. Wang *et al.*^137^ explored the effect of MoS_2_ on the fibrillation process of Aβ fragments and human islet amyloid polypeptide (hIAPP) fragments. A related study found that MoS_2_ allows for concentration-dependent modulation of amyloid aggregation. Mudedla *et al.*^138^ applied MD simulations to deeply study the interaction mechanism between amyloid fibrils and MoS_2_-based nanomaterials. MoS_2_-based nanomaterials cause the disruption of the secondary structure and change the β-sheet conformation to a flipped form. The results exhibited that the intermolecular force of peptides, including hydrophobic and hydrophilic interactions, was reduced due to the interaction between peptide and molybdenum disulfide materials. More destabilization of the fibril under nanotubes is observed compared to the nanosurfaces due to the difference in binding modes (Fig. 7A). Regrettably, no corresponding *in vivo* studies were performed. Liu *et al.*^139^ studied the effect of gold nanoparticle-doped molybdenum disulfide (AuNP-MoS_2_) nanocomposites on the aggregation of Aβ_40_. Low concentrations of AuNP-MoS_2_ can enhance the nucleation of Aβ_40_ and accelerate the aggregation of Aβ_40_. Although high concentrations of AuNP-MoS_2_ can enhance the nucleation of Aβ_40_ protein, it ultimately inhibits the Aβ_40_ aggregation process (Fig. 7B). It may be attributed to the interaction between AuNP-MoS_2_ and Aβ_40_ protein. A low concentration of AuNP-MoS_2_ can act as a nucleus. As the concentration of AuNP-MoS_2_ was increased, the structural transformation of the Aβ_40_ peptide was limited, leading to efficient inhibition of Aβ_40_ aggregation. MoS_2_ can rapidly heat up under NIR irradiation so that MoS_2_ can be used for photothermal therapy. Wang *et al.*^140^ designed multifunctional MoS_2_/AuNRs through the combination of MoS_2_ nanosheets and AuNRs. MoS_2_/AuNR can disrupt mature fibrils under NIR irradiation and prevent Aβ protein-induced neurotoxicity. It is worth mentioning that both MoS_2_ nanosheets and AuNRs can be used as NIR photothermal agents, and the MoS_2_/AuNR nanocomposites enhance the ability to destroy Aβ fibrils and enhance cell viability by generating localized heat under NIR irradiation (Fig. 7C). Because the specific cleavage sites of Aβ are often embedded in the β-sheet structure, artificial enzyme inhibition efficiency is severely hindered in practical applications. Qu's group constructs a NIR controllable artificial metalloprotease (MoS_2_-Co) using a MoS_2_ nanosheet and a cobalt complex of 1,4,7,10-tetraazacyclododecane-1,4,7,10-tetraacetic acid (Codota).^141^ MoS_2_-Co circumvented the β-sheet structural restrictions by simultaneous inhibition of the conformational switch from the random-coil to β-sheet structures and modulation of β-sheet structures of the preformed Aβ fibrils (Fig. 7D).

Li *et al.*^142^ found that WS_2_ nanosheets could effectively inhibit Aβ_40_ aggregation. Under van der Waals forces and electrostatic interactions, Aβ_40_ monomers can be selectively adsorbed on the nanosheet surface. WS_2_ has high NIR absorption properties, which can dissociate Aβ_40_ fibrils under NIR irradiation (Fig. 7E). Compared with traditional small molecular Aβ inhibitors, WS_2_ nanosheets can cross the BBB and exhibit excellent physicochemical characteristics.

The synthesis and modification methods of transition metal dichalcogenide nanosheets need to be further optimized. The preparation of nanosheets of specific thickness and size is essential. In addition, targeting issues and the biodegradation behavior of nanosheets need to be further explored.

### 3.5 Others

2D COFs, MXenes, hexagonal boron nitride and so on have also been reported for use in AD diagnosis and treatment.^120,143,144^

Covalent organic frameworks (COFs) are a new generation of nanoparticles consisting of carbon, oxygen, nitrogen and hydrogen atoms with excellent biocompatibility.^145^ 2D COFs have a highly tunable structure and can be designed to cross the blood–brain barrier and inhibit Aβ aggregation. Maleki *et al.*^143^ combined experimental and molecular simulation tools to investigate the interaction of novel two-dimensional COF materials with Aβ. The results indicate that amine-functionalized COFs with large surface areas have the potential to inhibit Aβ aggregation. Amine-functionalized groups were also found to enhance the ability of COFs to break the BBB. Two-dimensional transition metal carbides and/or nitriles (MXenes) possess a variety of enzyme-mimetic activities such as superoxide dismutase (SOD), catalase (CAT) and peroxidase (POD), which can be used for ROS scavenging against oxidative stress-induced inflammation and neurotoxicity. MXenes have good photothermal properties and improve the permeability of the BBB. Du *et al.*^120^ engineered 2D ultrathin Nb_2_C nanosheets to chelate metal ions and alleviate oxidative stress. *In vitro* experiments and theoretical calculations have demonstrated the antioxidant properties of Nb_2_C MXenzyme nanosheets and their specific chelating effect on Cu^2+^. In addition, the photothermal conversion properties of Nb_2_C MXenzyme nanosheets give them the ability to cross the BBB non-invasively.

Boron nitride nanomaterials have good chemical stability, antioxidant properties and biocompatibility. Unlike carbon nanomaterials, boron nitride nanomaterials are less hydrophobic and can maintain the conformation of Aβ rather than change it. Sorout *et al.*^146^ found that the interpeptide contacts are largely reduced in the presence of (3,3) boron nitride nanotube (BNNT) and that the nanoparticle interacts with the trimer in such a way that the initial helical secondary structure of the Aβ peptide is retained. The effect of different curvatures of boron nitride on Aβ aggregation was then continued to be investigated. And it was found that the planar boron nitride nanosheet (BNNS) with zero curvature is found to prevent β-sheet formation by converting the secondary structure of the peptide to dominant coil and turn conformations.^144^ The total number of peptide-nanoparticle contacts increases with a decrease in the curvature and a corresponding increase in the nanoparticle surface area. In addition, boron nitride nanoparticles have been reported as nanocarriers/agents to ameliorate Aβ-induced cytotoxicity.^147,148^ Currently for boron nitride nanomaterials differences from carbon nanomaterials have been revealed. Further research is expected to lead to a new generation of AD therapeutic nano-agents. Table 1 lists the mechanism and effect of two-dimensional inhibitors on the modulation of amyloid aggregation.

**Table 1:** A list of two-dimensional inhibitors for the modulation mechanism and effect of amyloid aggregation

Nanomaterials	Modulation mechanism	Effect	Ref.
GO	Adsorption/size effect	Delay	85
GO–ThS	Photothermal	Disaggregation	91
GOIO	Adsorption	Inhibition	84
GO@Dau	Adsorption/anti-oxidation	Inhibition/disaggregation	94
g-C_3_N_4_	Chelation	Inhibition/disaggregation	108
g-C_3_N_4_@Pt	Noncovalent interactions/platinum coordination/photooxygenation	Inhibition/disaggregation	105
Au/g-C_3_N_4_	Photooxygenation	Disaggregation	104
GO/g-C_3_N_4_	Photooxygenation	Disaggregation	106
g-C_3_N_4_	Photooxygenation	Inhibition	107
BP	Adsorption	Regulate the aggregation	118
PEG-LK7@BP	Electrostatic/hydrophobic interactions	Inhibition	119
BP@BTA	Photooxygenation	Inhibition	126
MoS_2_	Adsorption	Modulation	137
MoS_2_-Co	Photothermal	Inhibition/disaggregation	141
MoS_2_/AuNR	Photothermal	Modulation/disaggregation	140
AuNPs-MoS_2_	Concentration	Acceleration/inhibition	139
WS_2_	Photothermal/van der Waals/electrostatic interactions	Inhibition/disaggregation	142
2D COFs	van der Waals/electrostatic interactions/hydrogen bonds	Acceleration/inhibition	143
MXene	Chelation	Reducing ROS levels	120
BNNS	Adsorption	Modulation	144

## 4 Zero-dimensional nanomaterials

Zero-dimensional (0D) nanomaterials, including gold nanoparticles (GNPs), gold nanoclusters, organic and inorganic quantum dots, metal oxide nanoparticles, and carbon-based nanomaterials, have attracted extensive research interest in the field of biomedicine in recent years.^149^ The edge effect, quantum confinement effect, ultra-small size and good biocompatibility of 0D nanomaterials endow them with many functions and special performance, such as photoluminescence (PL), tissue penetration, bioactivity, and drug loading capability.^149^ Therefore, various 0D nanomaterials have been applied to diagnose and treat diseases, such as neurodegenerative disease, cancer and infection.^150^ Moreover, some advanced 0D nanomaterials can overcome the BBB and inhibit AD-related amyloid aggregation, so they are utilized to treat Alzheimer's disease.^151–153^ In this part, we summarized diverse treatment methods for amyloid and related neurodegenerative diseases by using different 0D nanomaterials.

### 4.1 Gold nanoparticles

**Fig. 8:**
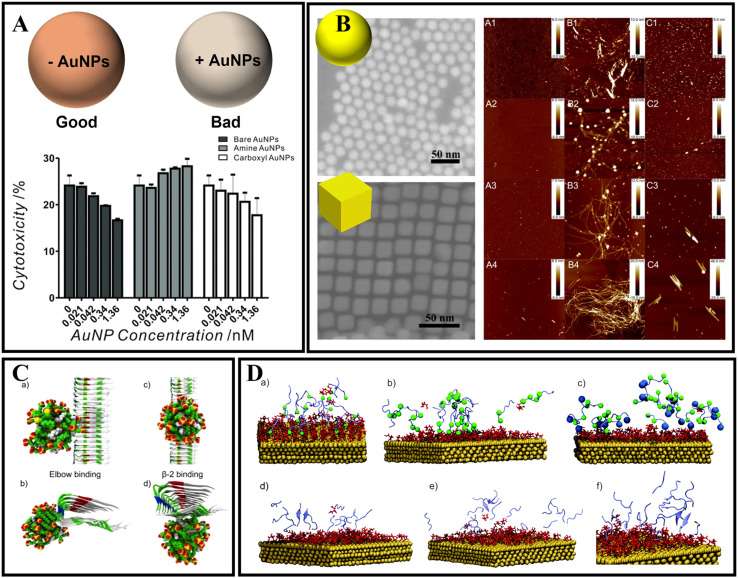
(A) Cytotoxicity of the end-point products of Aβ fibrillization incubated with and without bare, amine-conjugated, and carboxyl-conjugated AuNPs. Bare and negatively charged AuNPs both could effectively inhibit Aβ fibrillization and disaggregate Aβ fibrils and spherical oligomers compared with positively charged AuNPs.^157^ (B) AFM images (5 × 5 μm^2^) of the aggregates of Aβ_40_ (A1–4), Aβ_40_ and AuNS (B1–4), and Aβ_40_ and AuNC (C1–4) systems at different incubation times: 12 h (A1, B1, and C1), 24 h (A2, B2, and C2), 48 h (A3, B3, and C3), and 72 h (A4, B4, and C4).^158^ (C): (a) and (b) the elbow binding of a 2 nm 70% MUS-30% OT AuNP on the protofibril; (c) and (d) the β-2 binding as seen from the top and front of the fibrils.^160^ (D) Snapshots of MD simulations of amyloid peptides (purple) and gold surfaces (gold) covered with a citrate layer (red). (a) GNNQQNY peptide monomers bound to the gold surface. The terminal glycine (green ball) illustrated the favored N-terminal binding of the peptide to the citrate-stabilized gold nanoparticle surface. (b) NNFGAIL peptide monomers bound to the citrate-stabilized gold surface with the asparagine residues shown as green balls (N-terminus and position 2) to illustrate the N-terminal binding of the peptide. (c) VQIVYK peptide monomers (valine residues at the N-terminus and position 5 shown as green balls) at the gold surface with the C-terminal lysine (blue ball). The positively charged lysine at the C-terminus leads to binding of the peptide to the surface *via* both the N-terminus and the lysine side chain. The peptide monomers (VQIVYK) form parallel (d and e) and antiparallel (f) aligned dimers in solution and after binding to the gold surface.^161^

Gold nanoparticles (AuNPs) have attracted great interest as a novel platform in catalysis, drug delivery, and disease diagnosis/treatment owing to their biocompatibility, intriguing optical properties, surface functionalization, and immunological properties.^154,155^ Also, due to the diverse sizes, shapes, and surface properties, AuNPs have also been constructed to treat diverse central nervous system diseases. Moreover, AuNPs have been applied to modulate AD-related Aβ fibrillation under intracellular/extracellular spaces.^156^ Liao *et al.*^157^ studied the surface charge of AuNPs by different surface functionalization modifications for effecting Aβ fibrillation. Interestingly, although bare and negatively charged AuNPs both could effectively inhibit Aβ fibrillization and disaggregate Aβ fibrils and spherical oligomers compared with positively charged AuNPs, the negatively charged AuNPs exhibited higher inhibition ability than bare AuNPs during Aβ fibrillization-reduced neurotoxicity. Moreover, the neurotoxicity decreased only when incubated with bare and negatively charged AuNPs in a concentration-dependent manner (Fig. 8A). Apart from that, Wang *et al.*^158^ also studied the different shapes and effects of AuNPs on the aggregation of Aβ. The authors firstly prepared gold nanospheres (AuNSs) and gold nanocubes (AuNCs). The results of thioflavin T fluorescence assay showed that both AuNSs and AuNCs could inhibit Aβ fibrillation, but the effect efficiency of AuNSs is stronger than that of AuNCs. As shown in Fig. 8B, the shape of AuNPs influences the fibrillation kinetics of Aβ and the morphologies of Aβ fibrils. As a possible mechanism of shape-dependent AuNP–Aβ interactions, the authors analyzed that the surface energy of AuNPs is key for driving interaction between peptides and NPs. The AuNPs with an enormous specific surface area will inevitably adsorb peptide molecules on their surface. Compared to AuNCs, the spherical surface produces a large density of low-coordinated atoms situated on the edges and corners of AuNSs. Therefore, AuNSs have a stronger interaction with Aβ than AuNCs. Coincidentally, Tapia-Arellano *et al.*^159^ also found that the shape of the AuNPs could affect the aggregation kinetics of Aβ. They researched the effect of flat gold nanoprisms (AuNPr) and curved gold nanospheres (AuNSs) on Aβ aggregation kinetics and found that AuNPr accelerated the aggregation process and AuNSs slow down this process.

The interaction mechanism between the surface of gold nanoparticles and Aβ fibrils also needs to be studied with MD simulations. As shown in Fig. 8C, the AuNPs can interact with the amino-acid sequence of ^31^IIGLMVGGVVI^41^.^160^ After 10 ns, the AuNPs can move along the region of the β-sheet. Amino acids including Ile31, Gly33, Met35, Gly37, Val39, and Ile41 in Aβ fibrils were involved in binding with AuNPs. John's group also investigated the influence of AuNPs on peptide aggregation by studying the amyloid model peptides (Fig. 8D).^161^ They designed citrate-modified AuNPs and used MD simulations to confirm the structure-forming properties of the citrate-gold surface. They found that peptide monomers presented favored N-terminal adsorption to the surface of citrate-modified AuNPs by electrostatic attraction. Based on MD simulations, it was concluded that the initial contact of charged groups with the gold surface resulted in a local elevation and alignment of peptide monomers on the surface.

Besides studying citrate-modified AuNPs, biomolecular functionalized AuNPs have also been investigated. *Scutellaria barbata* leaf extract mediated AuNPs and mimosine functionalized AuNPs have also been identified to suppress AD-related β-amyloid aggregation and neuronal toxicity.^162,163^ However, the interactions between AuNPs and Aβ are typically nonspecific, and thus it is a great challenge to specifically target Aβ by using AuNPs. In addition, most studies have only focused on the simple surface–interface interactions between Aβ and AuNPs, the potential function needs to be deeply tapped. Therefore, Xiong *et al.*^164^ designed a kind of dual peptide coupled AuNPs. As one of the functional peptides, the VVIA (Aβ_39–42_) fragment can specifically target Aβ and efficiently reduce Aβ-induced toxicity by generating nontoxic heterooligomers. Meanwhile, LPFFD can efficiently interact with the KLVFFAE of the central hydrophobic cluster of the Aβ sequence. As a result, the inhibition ability of the corresponding peptide@AuNPs against Aβ aggregation and cytotoxicity is greatly improved. Thereafter, the dual peptide modified AuNPs (VVIACLPFFD (VCD10)@AuNP) are the most effective in inhibiting Aβ oligomerization and the cytotoxicity caused by the aggregation species.

### 4.2 Gold nanoclusters

Unlike AuNPs, gold nanoclusters (AuNCs) with a core size below 2 nm consist of a few to several hundred Au atoms.^165^ Thanks to their unusual properties, including strong photoluminescence, significant Stokes shift, good biocompatible, and biodegradation characteristics, AuNCs have been applied to disease-related diagnosis and treatment.^165^ Especially as an innovative nanomedicine, AuNCs also have significant promise in amyloid-related disease applications.

**Fig. 9:**
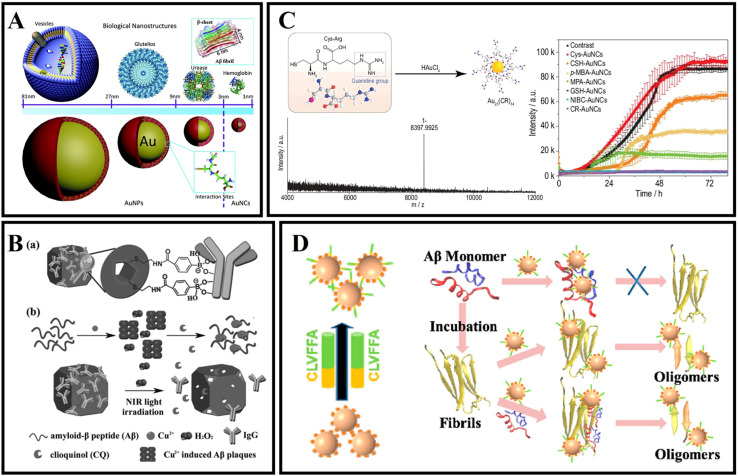
(A) Biomolecule-modified AuNPs and AuNCs to simulate different size biological entities to study the size effect of bio-nanointerfaces when they interact with Aβ.^166^ (B): (a) Illustration of IgG capped AuNC (AuNC-IgG). (b) H_2_O_2_-fueled and photothermal-responsive release of CQ from AuNC-IgG. CQ can chelate Cu^2+^ to disaggregate amyloid-β peptide (Aβ) plaques and inhibit H_2_O_2_ production.^167^ (C) Synthesis of CR-AuNCs and characterization of CR-AuNCs by ESI-MS and fibrillation kinetics for 20 μmol L^−1^ Aβ_40_ in the absence or presence of 25 mg L^−1^ Cys-AuNCs, CSH-AuNCs, *p*-MBA-AuNCs, MPA-AuNCs, GSH-AuNCs, NIBC-AuNCs or CR-AuNCs.^168^ (D) AuNCs-CLVFFA inhibited Aβ_40_ aggregation and prolongation, and disaggregated mature fibrils.^169^

As shown in Fig. 9A, Gao *et al.*^166^ reported nanoclusters (AuNCs) for the inhibition of amyloid aggregation. The authors prepared l-glutathione stabilized AuNCs and found that AuNCs with smaller sizes could completely inhibit amyloid aggregation and efficiently prevented Aβ from aggregation to larger oligomers, thus avoiding nucleation to form fibrils. As shown in Fig. 9B, Shi *et al.*^167^ designed a novel dual-responsive “cage metal chelator” release system based on AuNCs for non-invasive remote control to promote clioquinol (CQ) release and solubilize Aβ deposition. As a redox- and temperature-sensitive molecule, arylboronic esters were utilized to modify AuNCs for functionalized AuNCs. Therefore, the arylboronic ester-modified AuNCs could serve as a delivery system for H_2_O_2_-responsive controlled release. In addition, AuNCs possess a high near-infrared absorption and can further enhance the release of chelators under NIR light. As a result, this system can effectively inhibit Aβ aggregation and protect neurons from Aβ-reduced toxicity. Moreover, the photothermal effect of AuNCs can also serve as an effective means to dissolve Aβ amyloid deposits. Zhang *et al.*^168^ reported one type of Cys–Arg (CR) dipeptide modified Au nanocluster (Au_23_(CR)_14_) that was able to effectively dissolve pre-formed Aβ fibrils into monomers and recover the natural unfolded state of Aβ peptides from misfolded β-sheets (Fig. 9C). In addition, Au_23_(CR)_14_ was able to cross the BBB and cleared endogenous Aβ plaques in the brain of transgenic AD model mice. However, the interactions between traditional AuNCs and Aβ are also typically nonspecific, and thus it is also a great challenge to specifically target Aβ by using AuNPs. Recently, Hao *et al.*^169^ used a peptide fragment (CLVFFA) to modify AuNCs (AuNCs-CLVFFA) and CLVFFA could target binding the central hydrophobic region LVFFA of Aβ (Fig. 9D). Because the LVFFA is the central hydrophobic fragment of Aβ and can inhibit the aggregation of Aβ, AuNCs-CLVFFA was able to effectively inhibit Aβ aggregation and prolongation and disaggregate mature fibrils. Moreover, AuNCs-CLVFFA inhibited the transformation of Aβ from a random coil to a β-sheet structure.

We can also imagine the future development of functionalized AuNCs for amyloid aggregation-related diseases. With the deepening of research, we expect versatile AuNCs to become an essential platform for AD research.

### 4.3 Metal oxide nanoparticles

Metal oxide nanoparticles such as CeO_2_ NPs, ZnO NPs, CuO NPs, and Fe_3_O_4_ NPs have a variety of functional properties such as UV-barrier, antimicrobial, antioxidative, catalytic, and magnetic properties.^170,171^ Therefore, they have been extensively used in the field of drug delivery, disease diagnosis, disease treatment, and enzyme immobilization.^172^ Among them, CeO_2_ NPs, ZnO NPs, and Fe_3_O_4_ NPs have also been researched in amyloid aggregation-related neurodegenerative disorders.

**Fig. 10:**
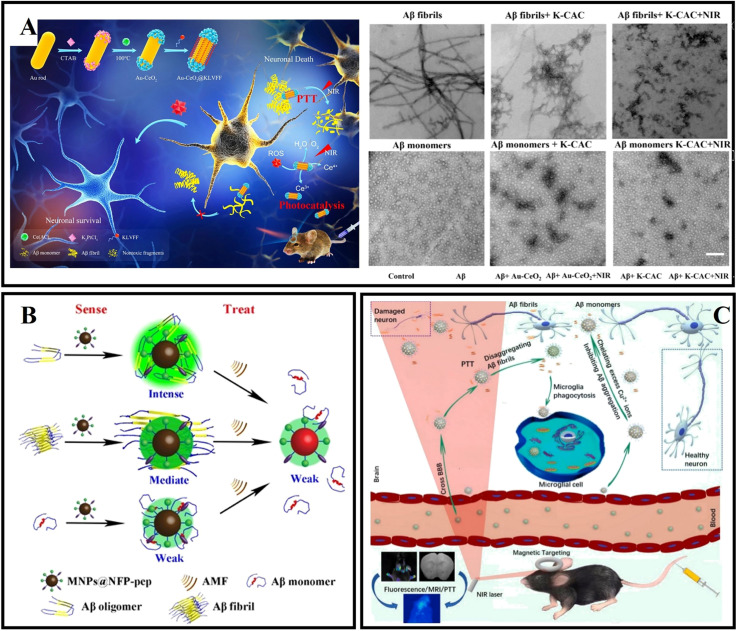
(A) Au-CeO_2_ exert antioxidant stress and target inhibition of Aβ through photocatalysis and the photothermal effect.^176^ (B) MNP@NFP-pep-based “sense and treat” system.^180^ (C) The B-FeCN nanosystem as a multifunctional nanocaptor with high BBB permeability to capture superfluous Cu ions and inhibit Aβ aggregation for magnetic targeting phototherapy.^18^

Due to their nontoxic nature, excellent biocompatibility and significant antioxidant activity at physiological pH values, cerium oxide nanoparticles (CeO_2_ NPs) have been given special attention.^173^ In addition, CeO_2_ NPs have both superoxide dismutase (SOD) mimetic activity and catalase mimetic activity by the Ce^3+^/Ce^4+^ valence transition, which also provides CeO_2_ NPs with an extra antioxidant function.^174^ Recently, CeO_2_ NPs have been used to protect neuron cells from Aβ-induced damage and treat neurocentral disease. In addition, CeO_2_ NPs can cross the BBB. Therefore, CeO_2_ NPs can be a promising candidate for treating AD. Recently, Li *et al.*^173^ designed a novel double delivery platform, which combined the advantages of controlled-release systems with those of glucose-coated CeO_2_ NPs (G-CeO_2_NPs). G-CeO_2_NPs could specially release the CeO_2_NPs and Cu^2+^ chelators by H_2_O_2_ stimulation. Therefore, the G-CeO_2_ NPs possess anti-aggregation properties and anti-oxidation properties. In addition, Li *et al.* adopted mesoporous silica nanoparticles as the carrier vehicles for loading G-CeO_2_NPs and 5-chloro-7-iodo-8-hydroxyquinoline. The research result showed that G-CeO_2_NPs could effectively inhibit Aβ aggregation, decrease cellular ROS and protect neurons from Aβ-induced toxicity. Guan *et al.*^174^ designed a bifunctional nanozyme (namely CeONP@POMs) by coating CeONP with POMs. The authors found that CeONP@POMs effectively inhibited Aβ aggregation, degraded Aβ aggregates, and reduced ROS levels. Moreover, CeONP@POMs is able to cross the BBB, regulate microglia, and protect neuronal cells from Aβ-related cytotoxicity. Coincidentally, a multifunctional AD therapeutic system, namely CeNP@MnMoS_4_, was designed and used to maintain metal ion homeostasis, reduce oxidative stress levels, and promote cell differentiation.^175^ Furthermore, due to the SOD activity, CeNP@MnMoS_4_ can protect cells from oxidative stress. Based on the catalase and superoxide dismutase activity of CeO_2_ and the hot electrons produced by gold nanorods, Ge *et al.*^176^ designed dumbbell-shaped nanocomposites (Au-CeO_2_) by coating both ends of gold nanorods with CeO_2_ NPs, and endowed Au-CeO_2_ with photocatalysis and photothermal effects in the NIR (Fig. 10A). To further improve the therapeutic efficiency of Au-CeO_2_, the authors used Aβ-targeted peptides (KLVFF) to modify Au-CeO_2_ and obtained an Aβ-targeted nanocomposite (K-CAC). The related results exhibited that K-CAC could improve the cognitive function of AD mice.

As a type of magnetic nanoparticles (MNPs), iron oxide nanoparticles (IONs) are considered promising materials due to their high biocompatibility, unique magnetic properties, and ability to function as multimodal contrast agents.^177,178^ In addition, IONs have potential high affinity for circulating Aβ forms to induce a “sink effect” and potentially ameliorate AD.^179^ Mahmoudi *et al.*^178^ found that lower concentrations of superparamagnetic iron oxide nanoparticles (SPIONs) inhibited fibrillation, while higher concentrations increased the rate of Aβ fibrillation. And it was evident that the positively charged SPIONs could promote fibrillation compared with negatively charged or uncharged SPIONs. Currently, the surface functionalization of nanoparticles by using chemical methods is becoming more and more popular. Qu's group designed a multi-functional nanosystem (MNP@NFP-pep) by modifying a naphthalimide-based fluorescent probe and KLVFF peptide on the surface of magnetic nanoparticles, which can both specifically detect Aβ oligomers and achieve the wireless deep magnetothermally mediated disaggregation of Aβ aggregates with an alternating magnetic field.^180^ MNP@NFP-pep can interact with the exposed hydrophobic residues of Aβ oligomers based on π–π stacking and hydrophobic interaction (Fig. 10B). MNP@NFP-pep was able to specifically target Aβ aggregates and break down Aβ aggregates. Recently, our group presented drug-based magnetic imprinted nanoparticles (MINs@EGCG) combined with epigallocatechin-3-gallate (EGCG) and magnetic nanoparticles.^19^ MINs@EGCG exhibited triple functions for amyloid inhibition, drug delivery and fiber separation under an external magnet. MINs@EGCG inhibited the formation of amyloid fibrils with a high efficiency for 80%. Moreover, with the help of an external magnetic field, the cleaning efficiency is up to 80%. In addition, Halevas *et al.*^181^ prepared a nanocarrier (MMSNPs) by the sol–gel method using a magnetic core of Fe_3_O_4_ and a mesoporous silica shell and modified the flavonoid quercetin on the surface of MMSNPs for obtaining QCMMSNPs. QCMMSNPs exhibited potential anti-amyloid and antioxidant abilities. Moreover, QCMMSNPs reduced Aβ-induced cellular toxicity and minimized Aβ-induced ROS generation. Recently, Dyne *et al.*^182^ found that mild magnetic nanoparticle hyperthermia could destroy mature Aβ fibers by local heat and facilitate the phagocytic clearance of Aβ as well as attenuating pro-inflammatory responses by microglial cells. As shown in Fig. 10C, Gong *et al.*^18^ reported an intelligent nanosystem (B-FeCN) by modifying carbon nitride nanodots and benzothiazole aniline on the surface of Fe_3_O_4_@mesoporous silica nanospheres. Among them, B-FeCN effectively traps excessed Cu^2+^ and inhibits the formation of Cu^2+^–Aβ complexes. In addition, B-FeCN generated local heat to promote the depolymerization of fiber precipitates. Interestingly, the BBB permeability of B-FeCN was significantly improved under NIR irradiation. Thanks to the advantages of the Fe_3_O_4_ cores, B-FeCN entered the brain and targeted the Aβ region with the help of a magnetic field. Benzothiazole aniline (BTA) makes B-FeCN a detection agent for specifically targeting Aβ plaques and imaging the Aβ species by fluorescence. However, B-FeCN has a certain biological toxicity, and the research on the metabolic mechanism *in vivo* is not perfect, which hinders further applications.

### 4.4 Organic and inorganic quantum dots

Therapeutic agents should be completely cleared from the body in a reasonable time, and usually, effective renal and hepatic clearance requires drugs less than 10 nm, and the development of nanoparticles with excellent biocompatibility is of great importance.^183^

**Fig. 11:**
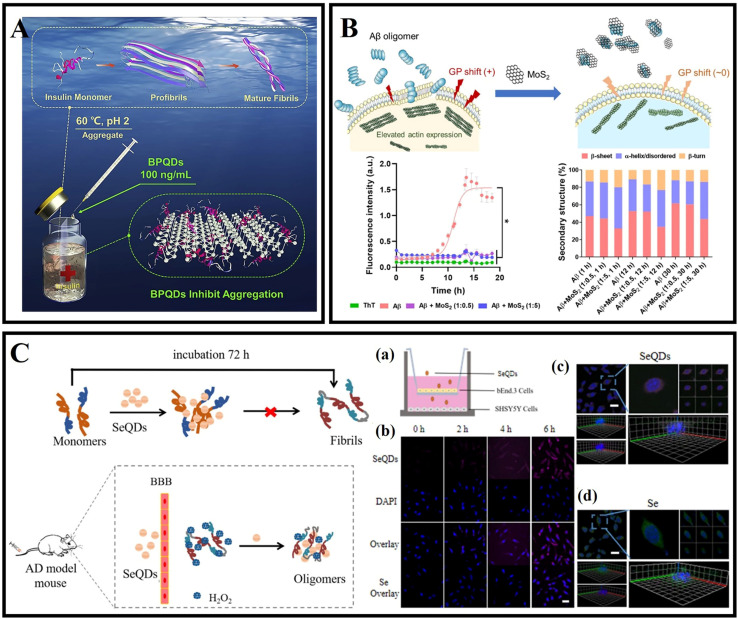
(A) Amyloid aggregation of insulin in the absence and presence of BPQDs.^185^ (B) Cell membrane disruption by Aβ oligomers and its rescue by ultrasmall MoS_2_ QDs. ThT fluorescence kinetic assay and attenuated total reflection (ATR)-FTIR indicated the inhibitory effects of ultrasmall MoS_2_ QDs on Aβ.^190^ (C) Se QDs for dissociating Aβ fibrils and crossing the BBB. (a and b) Transwell experiment and cellular uptake. Scale bar, 25 μm. (c and d) 3D SH-SY5Y cell fluorescence image. Scale bar, 30 μm.^194^

Sun *et al.*^184^ prepared BPQDs with excellent NIR photothermal properties and biocompatibility using the liquid phase exfoliation method. The size distribution of the prepared BPQDs was only 2.6 nm. BPQDs were conjugated with PEG and exhibited high stability in the physiological medium and low toxicity for different cell types. More importantly, BPQDs induced the death of C6 and MCF7 cancer cells under NIR illumination, indicating that the BPQDs have great potential as photothermal agents with implications for the treatment of amyloid-related diseases. Wang *et al.*^185^ found that BPQDs at 100 ng mL^−1^ inhibited insulin aggregation and disaggregated mature fibers, and the inhibitory effect persisted through all stages of insulin aggregation (Fig. 11A). Molecular dynamics simulations showed that BPQDs could stabilize the α-helix structure of insulin and reduce the β-sheet content. Bu *et al.*^186^ reported using BPQDs as a photoactive material and heme as an electron acceptor sensor to monitor the Aβ protein content, and these properties make BPQDs a promising candidate for the treatment of amyloidosis and neurodegenerative disease.

Molybdenum disulfide quantum dots (MoS_2_ QDs) have been widely used for live bioimaging and nanomedicine because of their low toxicity, excellent cell permeability and biocompatibility, and strong luminescence properties.^187,188^ Sun *et al.*^189^ used a one-pot hydrothermal method to synthesize cysteamine functionalized MoS_2_ QDs, which effectively inhibited the fibrillation and destabilized preformed fibrils of bovine serum albumin in a concentration-dependent manner. Li *et al.*^190^ observed cell membrane perturbation and actin reorganization, which were induced by Aβ oligomers. Further research revealed that the ultra-small MoS_2_ QDs restored membrane fluidity and inhibited Aβ amyloid aggregation (Fig. 11B). Based on the calculation of discrete molecular dynamics simulations, it was found that MoS_2_ QDs were bound to the N-terminal of Aβ peptides through hydrophilic interactions. In addition, surface-coated Aβ oligomers by MoS_2_ QDs could not further associate with cell membranes. Tian *et al.*^191^ pointed out the promising application of MoS_2_ QDs in photodynamic therapy. MoS_2_ QDs promote the creation and separation of electron–hole pairs more effectively than MoS_2_ nanosheets. Therefore, MoS_2_ QDs are able to generate a variety of ROS under illumination. Results related to MoS_2_ QDs broaden the application of molybdenum disulfide-based nanomaterials.

As drugs or nanocarriers, selenium nanoparticles have made important progress in cancer, AD and other diseases because of their excellent physicochemical characteristics.^192^ It has been reported that selenium nanoparticles have a high affinity for Aβ, which can inhibit Aβ aggregation and treat AD as a potential nanomedicine.^193^ As shown in Fig. 11C, Guo *et al.*^194^ synthesized selenium quantum dots (Se QDs), which could quickly penetrate the BBB because of their ultrasmall size and excellent biocompatibility. Se QDs had a strong free-radical scavenging activity and could protect cells from oxidative stress damage. Se QDs could not only inhibit Aβ aggregation and reduce Aβ-mediated cytotoxicity, but also effectively reduce tau protein phosphorylation, further improve oxidative stress, and maintain nerve cell stability. In conclusion, Se QDs had great advantages compared with traditional single-target drugs in the treatment of AD.

### 4.5 Carbon-based zero-dimensional nanomaterials

Carbon nanomaterials are widely used to inhibit Aβ aggregation due to the various surface and interface interactions between the Aβ peptide and carbon nanomaterials.^34^ Carbon dots (CDs), as a new type of carbon-based zero-dimensional nanomaterial, have attracted extensive research in recent years because of their low cost, easy synthesis, good biocompatibility, photoluminescence, easy surface modification, and high stability.^195^ It's important to note that CDs include graphene quantum dots (GQDs), carbide polymer dots (CPDs), and carbon quantum dots (CQDs).^196^

**Fig. 12:**
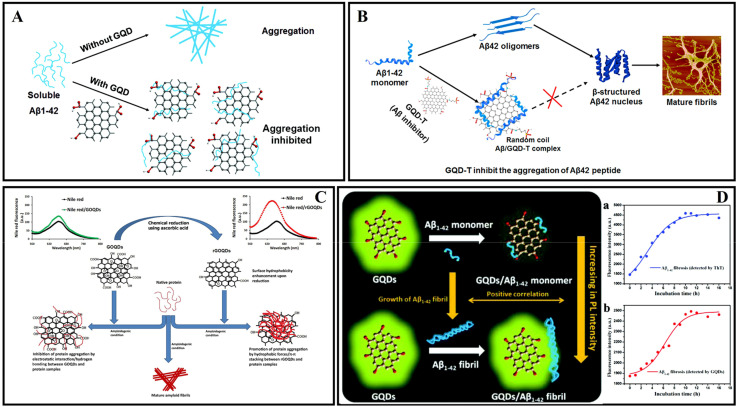
(A) The GQDs used for inhibiting the aggregation of Aβ_42_ peptides.^199^ (B) GQD-T inhibits the aggregation of Aβ_42_ peptides.^203^ (C) GOQDs have the capacity to inhibit the fibrillation of protein.^208^ (D) Proposed mechanism for the different fluorescence behaviors of GQDs on Aβ_42_ monomers and fibrils.^209^

GQDs are single- or few-layered graphene sheets of 10 nm or less in size.^197^ Most CDs possess size-dependent auto-fluorescence originating from quantum confinement and edge effects, compared with carbon nanotubes, fullerenes, and graphene nanomaterials.^198^ According to previous studies, GQDs have a good ability to cross the BBB, effectively modulate the Aβ aggregation process and reduce Aβ-induced neurotoxicity.^95^ Therefore, GQDs are often combined with Aβ aggregation inhibitors or neuroprotective peptides to enhance efficacy. In 2015, Liu *et al.*^199^ prepared GQDs by a hydrothermal method, demonstrating that GQDs effectively inhibited Aβ_42_ peptide aggregation (Fig. 12A). Moreover, Xiao *et al.*^200^ prepared a novel nanomaterial GQDG by conjugating GQDs with glycine–proline–glutamate (Gly–Pro–Glu). *In vitro* assays proved that both GQDs and GQDG could inhibit the aggregation of Aβ_42_. *In vivo* assays indicated that GQDG enhanced AD model mice's learning and memory capacity, increased dendritic spine amounts, and decreased several pro-inflammatory cytokine content. Subsequently, several studies reported the application of nitrogen-doped graphene quantum dots (N-GQDs) and fluorine-functionalized graphene quantum dots (FGQDs) in amyloid aggregation.^201,202^ Liu *et al.*^203^ covalently combined GQDs with tramiprosate to design a novel Aβ aggregation inhibitor, namely GQD-T. GQD-T showed the capability of inhibiting Aβ aggregation and rescuing Aβ-induced cytotoxicity due to the synergistic effect of the GQDs and tramiprosate (Fig. 12B). Moreover, GQDs can effectively disperse mature amyloid-rich *Staphylococcus aureus* biofilms and interfere with the self-assembly of amyloid fibers.^204^ Liu *et al.*^205^ studied the regulatory effects and mechanism of GQDs on Aβ_42_ aggregation and found that electrostatic interaction was the major driving force in the co-assembly process of Aβ_42_ and GQDs. Tak *et al.*^206^ used *Clitoria ternatea* as a precursor with the help of a one-pot microwave-assisted method to prepare novel graphene quantum dots ctGQDs. The transport efficiency of ctGQDs across the BBB was increased significantly and showed high inhibition efficiency of the acetyl cholinesterase enzyme. Meanwhile, Perini *et al.*^207^ reviewed the potential of GQDs in biomedicine and neuroscience and discussed the ability of GQDs to cross the BBB and reach the brain. Ghareghozloo *et al.*^208^ studied the inhibiting effect of graphene oxide quantum dots (GOQDs) on bovine insulin and hen egg white lysozyme (HEWL) aggregation. GOQDs were prepared through pyrolysis of citric acid, and the reduction step was carried out using ascorbic acid. The results showed that GOQDs could inhibit the related protein fibrillation, and the presence of reduced GOQDs was found to promote protein assembly *via* shortening the nucleation phase. The content of oxygen-containing functional groups from the GOQD surface may be the key factor in affecting fibrillation (Fig. 12C).

The detection of the concentration of amyloid monomer is of great importance in diagnosing AD. Huang *et al.*^209^ proposed a method to detect Aβ monomer concentration using the fluorescent properties of GQDs (Fig. 12D). The positively charged groups, the aromatic structure and moieties with hydrogen bonding ability on Aβ_42_ monomers provided suitable conditions for the interaction between Aβ_42_ monomers and GQDs. This strong combination promoted the excited-state electron transfer from GQDs to Aβ_42_, resulting in quenching of the PL intensity of GQDs. The Aβ fibers consume abundant interaction sites and contact surface areas through a self-assembly process, and the interaction between Aβ fibers and GQDs is much weaker to quench GQD fluorescence. Yousaf *et al.*^210^ reported the detection of monomers and oligomers using specific fluorescence and a magnetic resonance imaging (MRI) multimodal probe based on bovine–serum–albumin-capped fluorine functionalized GQDs (BSA@FGQDs). BSA@FGQDs could monitor amyloid fibrillation and was more sensitive than conventional ThT stain. Monitoring amyloid aggregation dynamics and monomers/oligomers using BSA@FGQD probes is based on hydrophobic, electrostatic, hydrogen bonding, and π–π stacking interactions. Tang *et al.*^211^ examined the influences of GQDs on the obstruction of the membrane axis of Aβ in its three forms of monomers (Aβ-m), oligomers (Aβ-o), and amyloid fibrils (Aβ-f), and demonstrated the mitigation potential of GQDs in reverting SH-SY5Y cells to their native fluidic state. It was found that Aβ-m is bound to the GQDs *via* strong electrostatic and hydrophobic interactions. The nanostructures reshaped the potential of mean force (PMF) of Aβ-o to inhibit the β-sheet propensity of the peptide residues, and GQDs adhered to the sides and ends of an Aβ-f, thereby hindering their elongation.

CQDs are a new class of 0D carbonaceous nanomaterials with a diameter less than 10 nm.^212^ CQDs can be produced using diverse bioorganic compounds through solvent-free pyrolysis, hydrothermal treatment, or microwave treatment. These treatment methods and bioorganic compounds allow for the synthetic flexibility of CQDs without intricate set-ups.^213^ CQDs have outstanding features such as low cost, easy synthesis, excellent biocompatibility, and photoluminescence.^212^ The absorption and emission spectra of CQDs can be tuned by adjusting the precursor type, preparation method, degree of carbonization, surface state, and element doping.^214^ In addition, CQDs have abundant functional groups, such as hydroxyl, amino, and carboxyl groups, which are easy to modify.^215^ Moreover, CQDs can interact with Aβ peptides and aggregates through electrostatic, hydrogen bonding, π–π stacking, and hydrophobic interactions.^15,216^

**Fig. 13:**
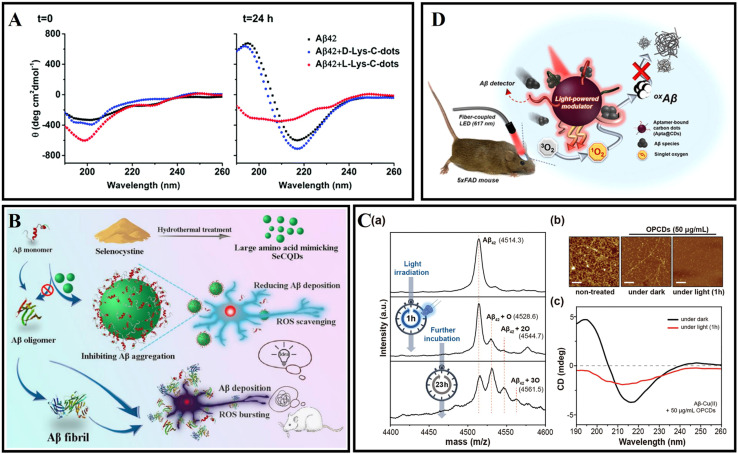
(A) Secondary structures of 25 mM Aβ_42_ monitored by CD spectroscopy in the absence or presence of d-Lys-C-dots or l-Lys-C-dots.^219^ (B) Synthesis of SeCQDs and the inhibition effects of SeCQDs on Aβ aggregation and ROS production.^221^ (C): (a) The mass spectra of Aβ–Cu(ii) aggregates incubated with OPCDs for 24 hours at 37 °C. Light was irradiated only for one hour. (b) AFM images and (c) CD spectra of Aβ–Cu(ii) aggregates incubated with OPCDs under dark and one-hour-light illuminating conditions.^232^ (D) Aβ-targeting, CD-mediated photodynamic modulation for spatiotemporal inhibition of Aβ aggregation *in vivo*.^229^

Many studies have reported the inhibition of human insulin fibrosis by using carbon dots.^217,218^ Malishev *et al.*^219^ prepared enantiomeric carbon dots (l-Lys-C-dots and d-Lys-C-dots) using l-lysine or d-lysine. The results demonstrated that l-Lys-C-dots exhibited higher affinity to Aβ_42_ (either monomeric and/or pre-fibrillar species) compared with d-Lys-C-dots, modulated the fibril assembly process of Aβ_42_ (Fig. 13A). The authors speculated that the different properties of l-Lys-C-dots and d-Lys-C-dots were caused by residual lysine moieties which exposed to the C-dots’ surface and residual lysine possibly interfered with the electrostatic interactions of the peptide. Zhou *et al.*^15^ used *o*-phenylenediamine and citric acid as precursors to synthesize amphiphilic yellow-emissive CDs (Y-CDs) by an ultrasonication-mediated methodology. The amphiphilicity of Y-CDs didn't change with different coatings. In addition, it was proved that Y-CDs could cross the BBB of zebrafish *via* passive diffusion. The related research suggested that Y-CDs could inhibit the overexpression of APP and Aβ peptides. Koppel *et al.*^220^ used brown coal to prepare novel CQDs. CQDs were able to inhibit IAPP and Aβ aggregation induced by lipopolysaccharide (LPS) through hydrogen bonding and hydrophobic interactions. This study contributed to understanding the pathological link between bacterial metabolites and amyloid diseases. Due to the excellent antioxidant capacity of selenium nanoparticles, Zhou *et al.*^221^ designed selenium-doped carbon quantum dots (Se-CQDs) *via* a simple hydrothermal treatment of selenocystine, which were successfully applied to inhibit Aβ aggregation and scavenge the redundant ROS in the brain (Fig. 13B). Se-CQDs maintained the intrinsic properties of both selenium and CQDs. Se-CQDs have paired α-carboxyl and amino groups at their edges, which trigger multivalent interactions with Aβ. Li *et al.*^222^ fabricated Se-CQDs using selenocysteine through hydrothermal treatment under mild conditions. ROS could be effectively scavenged by the Se-CQDs. Once Se-CQDs are internalized into cells, high levels of ROS in cells are reduced. These properties enable Se-CQDs to protect biological systems from oxidative stress. Guerrero *et al.*^223^ used Na-citrate as a precursor to prepare CQDs. Pulse-chase lysozyme fibril-forming assay and ThT fluorescence showed that CQDs prevented the monomers and oligomers into mature fibrils, while could provoke the disaggregation of mature HEWL fibrils. Li *et al.*^224^ fabricated ultra-small CQDs with a uniform size by pulsed laser ablation. Results demonstrated that CQDs could efficiently inhibit Aβ_42_ aggregation. Moreover, the quenching of tyrosine and ANS fluorescence of the Aβ_42_ solutions with CQDs indicated that there existed an interaction between the CQDs and Aβ_42_ peptides. Our group prepared glycosylated carbon dots (g-CDs) using glucose as a precursor. gCDs-E has been prepared by self-assembly of gCDs and epigallocatechin-3-gallate (EGCG). gCDs-E could not only suppress the fibrillation of Aβ and disaggregate Aβ fibrils, but also effectively inhibit the activity of *Candida albicans*.^13^ In addition, the capability of gCDs-E for BBB penetration was also observed using a normal mice model.

As a highly active substance, ROS can be used as a disease treatment agent.^225^ At present, there are many research studies about photodynamic therapy for tumor diseases.^226^ In addition, photodynamic therapy also has good application potential in amyloid-related diseases.^227^ The band-to-band transition of CDs' electron carriers generates ROS through an electron- (type I) or energy-transfer (type II) process, mediating photo-modulation to denature target biotoxins.^228,229^ Like a type II photosensitizer, CQDs can react with oxygen after absorbing energy, promote the production of singlet oxygen, and oxidize the amino acid residues of Aβ peptides, thereby destroying the interaction between peptides.^230^

For example, Chung *et al.*^231^ synthesized CDs using ammonium citrate through one-pot hydrothermal treatment and obtained branched polyethylenimine modified CDs (bPEI@CDs) by passivating the surface of the prepared CDs using branched polyethylenimine. bPEI@CDs exhibited hydrophilic and cationic surface properties, which could effectively interact with negatively charged residues of Aβ peptides. Under light illumination, bPEI@CDs displayed a strong effect on Aβ aggregation and on the disaggregation of fibrils by generating ROS. Building on previous work, Chung *et al.*^232^ prepared multifunctional carbon-dots (OPCDs) using *o*-phenylenediamine. The N-containing polyaromatic surface of OPCDs is the reason why the self-assembly of Cu(ii)-Aβ is hindered, thereby weakening Cu(ii) catalyzed oxidative stress and Aβ aggregation propensity. Illumination treatment further enhanced the inhibitory effect of OPCDs, which produced ROS to oxidize the key residues (His and Met) of Aβ (Fig. 13C). Recently, Chung *et al.*^229^ designed Aβ-targeted, red light-responsive apta@CD based on previous work, which inhibited Aβ aggregation in space and time and reduced the overall Aβ burden in the brain. Under red light, apta@CDs effectively inhibited the formation of Aβ aggregates by oxidizing Aβ residues, exhibiting a light-modulating effect on Aβ aggregation (Fig. 13D). The application of apta@CDs to 5xFAD mice further demonstrated the anti-amyloid aggregation ability of apta@CDs *in vivo*.

CPDs have also been reported for AD diagnosis and treatment. Gao *et al.*^233^ prepared multifunctional nitrogen-doped CPDs by using *o*-phenylenediamine for targeting Aβ aggregations. CPDs inhibited Aβ fibrillation and disaggregated Aβ fibrils through electrostatic interactions, hydrogen bonds, and hydrophobic interactions. CPDs could emit enhanced red fluorescence upon interaction with Aβ fibrils, clear amyloid plaques *in vivo* and prolong the lifespan of CL2006 strain by alleviating Aβ-induced toxicity. C_60_ has been demonstrated to interact and prevent Aβ fibrillation. However, there are significant problems such as low solubility and toxicity that need to be solved.

Fullerenes and their derivatives have been reported for use in amyloid diseases. Melchor *et al.*^234^ synthesized diethyl fullerenemalonates and the corresponding sodium salts using the Bingel reaction, adducts of C_60_ bearing 1 to 3 diethyl malonyl and disodium malonyl substituents (C_60+*n*_(COOR)_2*n*_, where *n* = 1–3 and R = –CH_2_CH_3_, –Na). The inhibition efficiency of bisadduct salts (C_62_(COONa)_4_) and trisadduct (C_63_(COONa)_6_) is 98% and 83% respectively. The 6.7 mM C_62_(COONa)_4_ mixture has been confirmed the capacities for anti-amyloid deposition. The anti-aggregation effect of C_62_(COONa)_4_ is mainly attributed to the hydrophobic surface and the number of substituents bound on the surface of fullerene. C_60_ acts as both a ROS producer under UV-visible light and a ROS scavenger in the dark.^235^ Du *et al.*^236^ designed UCNP@C_60_-pep nanoparticles, which generated ROS under NIR light and oxidized Aβ. Moreover, UCNP@C_60_-pep could also alleviate the excessive ROS in the organization. Both the ROS generation and ROS quenching abilities of UCNP@C_60_-pep were beneficial to reduce Aβ-induced neurotoxicity. Bobylev *et al.*^237^ studied the ability of water-soluble fullerene derivatives with different types of solubilizing addends for anti-amyloid aggregation. The three derivatives were found to exhibit strong anti-amyloid effects *in vitro* and low cytotoxicity *in vivo*. The fullerene derivatives have a strong anti-amyloid effect and low toxicity. Their ability for crossing the BBB and the inhibition ability of amyloid fibrillation make fullerenes potential drug candidates. Table 2 lists the mechanism and effect of carbon-based zero-dimensional nanomaterials on the modulation of amyloid aggregation.

**Table 2:** A list of carbon-based zero-dimensional nanomaterials with the modulation mechanism and effect of amyloid aggregation

Nanomaterials	Modulation mechanisms	Effects	Ref.
GQDs	Adsorption/hydrophobic interactions	Inhibition	199
GQDG	Adsorption	Inhibition	200
FGQDs	Adsorption	Inhibition/disaggregation	201
GQD-T	Adsorption/synergistic	Inhibition	203
ctGQDs	Inhibition acetyl cholinesterase enzyme	Treating disorganization of cells	206
GOQDs	Adsorption/hydrophobic interactions	Inhibition	208
BSA@FGQDs	Hydrophobic/electrostatic/H-bonded/π–π stacking interactions	Monitor	210
l-Lys-C-dots	Electrostatic interactions	Modulation	219
Y-CDs	Amphiphilic	Inhibiting overexpression of APP and Aβ peptides	15
CQDs	H-Bonded/hydrophobic interactions	Inhibition	220
Se-CQDs	Adsorption/hydrophobic interactions/anti-oxidation	Inhibiting Aβ aggregation and scavenging ROS	221
Se-CQDs	Anti-oxidation	Scavenging ROS	222
gCDs-E	Hydrophobic interactions/anti-oxidation	Suppressing fibrillation/disaggregation/inhibition fungi	13
bPEI@CDs	Electrostatic interactions/photooxygenation	Inhibition/disaggregation	231
OPCDs	Chelation/hydrophobic interactions/photooxygenation	Inhibition	232
Apta@CDs	Photooxygenation/target	Inhibition	229
CPDs	Electrostatic interactions/hydrogen bonds/hydrophobic interactions	Inhibition/disaggregation	233
C_60_	Hydrophobic interactions	Inhibition	234
UCNP@C_60_-pep	Photooxygenation/anti-oxidation	Inhibition/scavenging ROS	236

## 5 Others

In addition, other nanoparticles have also been reported for diagnosis and treatment of AD, including metal–organic frameworks (MOFs), polyoxometalates (POMs), liposomes, SiO_2_, upconverting nanoparticles and so on.^238–241^

### 5.1 Metal–organic frameworks

Metal–organic frameworks (MOFs) are crystalline entities composed of metal ions or clusters and polydentate organic ligands.^242^ As an emerging family of hybrid nanomaterials, MOFs have attracted much attention due to their porous structures, good biocompatibility, and tunable sizes, and are widely used in catalytic, sensing and biological applications.^243–246^

**Fig. 14:**
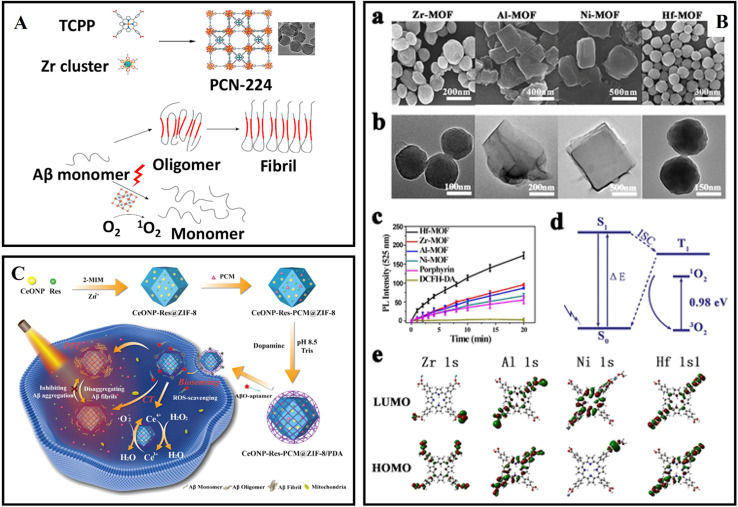
(A) The synthesis of PCN-224 nanoparticles and photo-inhibition of Aβ_42_ aggregation by using PCN-224 nanoparticles.^238^ (B): (a) SEM images. (b) TEM images of Zr-MOF, Al-MOF, Ni-MOF, and Hf-MOF, respectively. (c) Fluorescence intensity of DCF after photooxidation by using PMOFs at different time points. (d) Jablonski diagram describing the underlying photophysics and photochemistry of photodynamic therapy. (e) Electronic density contours for the frontier molecular orbitals of the four kinds of PMOFs.^247^ (C) CeONP-Res-PCM@ZIF-8/PDA preparation and its applications in Aβ oligomer sensing and treatment.^248^

Wang *et al.*^238^ prepared NIR responsive nanoparticles PCN-224 for inhibiting Aβ aggregation. PCN-224 was hydrothermally synthesized by coordinating tetrakis(4-carboxyphenyl)porphyrin (TCPP) ligands with zirconium (Fig. 14A). Under NIR irradiation, PCN-224 significantly reduced Aβ induced cytotoxicity. The functional porphyrin linkers are separated by Zr clusters in the MOF framework, which could avoid the self-quenching of excited states, maintain the photo-oxidative properties of the porphyrin linkers, and improve the ^1^O_2_ generation capacity. Yu *et al.*^247^ selected four kinds of POMFs (Zr-MOF, Al-MOF, Ni-MOF, and Hf-MOF) for further investigation, which are stable under physiological conditions and exhibit excellent biocompatibility (Fig. 14B). It was found that Hf-MOF was the most efficient Aβ photooxidant based on the experimental results and DFT calculations. LPFFD modified Hf-MOFs not only effectively targeted Aβ peptides and reduced Aβ-induced cytotoxicity, but also improved photooxidation in complicated environments. Yan *et al.*^248^ synthesized a core–shell nanocomposite CeONP-Res-PCM@ZIF-8/PDA/Apt through an *in situ* encapsulation strategy. Resveratrol (Res), ceria nanoparticles (CeONPs) and PCM (tetradecanol) were embedded in a ZIF-8/PDA matrix by a water-based mild method (Fig. 14C). These nanocomposites can be activated to release the encapsulated Res upon NIR illumination through PCM regulation. Moreover, CeONP-Res-PCM@ZIF-8/PDA/Apt nanocomposites exhibited multifunctional effects on inhibiting Aβ aggregation, disaggregating Aβ fibrils, and decreasing Aβ-induced oxidative stress and neural apoptosis. The therapeutic effect of nanocomposites could be enhanced under NIR irradiation because of the excellent photothermal properties of PDA. In 2021, Zeng *et al.*^249^ built an electron-deficient MOF from the ligand of naphthalene diimide (NDI) and metal nodes of biocompatible Ca^2+^. Then pyrene as an electron donor molecule was encapsulated to form a host–guest MOF self-assembled co-crystal Py@Ca-NDI. A concomitant superior charge transfer interaction between pyrene and NDI could be attained and the photothermal conversion efficiency of Py@Ca-NDI in aqueous solution could reach up to 41.8%. The treatment of neurodegenerative disease by using MOF-based materials is a challenging study, and more elaborative studies on biostability, biocompatibility and BBB penetration are still needed.^250^

### 5.2 Polyoxometalates

Polyoxometalates (POMs) are a special group of inorganic redox-active materials consisting of multiple metal oxide ions linked together by oxygen atoms to form nanoclusters within an ordered three-dimensional framework.^251^ Due to the tunable structures, excellent physicochemical properties and good biocompatibility of POMs, many researchers have explored their application in biomedicine. POMs can act as Aβ aggregation inhibitors and can be seen as candidates for the treatment of AD because of their similarity to water-soluble fullerene derivatives.^252^

**Fig. 15:**
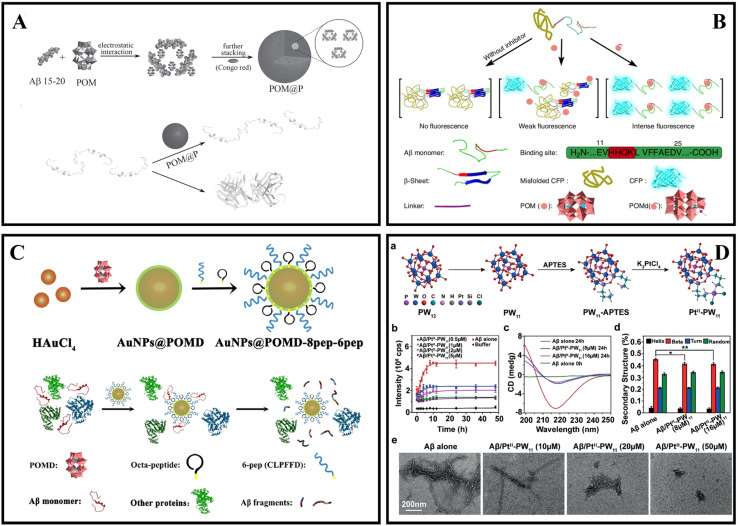
(A) The schematic illustration of self-assembly of Aβ_15–20_ and POM to hybrid spheres and the assembled peptide and POM nanoparticles can effectively inhibit Aβ_1–40_ aggregation.^254^ (B) The high-throughput screening method for identifying effective Aβ-aggregation inhibitors.^255^ (C) Synthetic route of the Aβ-targeted nanozyme and AuNPs@POMD-8pep-6pep acted as an Aβ targeted nanozyme to specifically hydrolyze Aβ.^256^ (D) Inhibition effect of Pt^II^-PW_11_ on Aβ_42_ aggregation. (a) The synthesis of Pt^II^-PW_11_. (b) Aggregation kinetics of Aβ_42_ without or with Pt^II^-PW_11_ monitored by ThT fluorescence assay. (c) CD spectra and (d) secondary structure analysis of Aβ_42_ without or with co-incubation of Pt^II^-PW_11_. (e) TEM images of Aβ_42_ without or with co-incubation of Pt^II^-PW_11_ at 37 °C for 48 h.^29^

In 2011, Qu's group reported the inhibitory effect of POMs on Aβ aggregation and found that POMs with a Wells–Dawson structure had a better effect.^239^ Then Li *et al.*^253^ reported that POMs could not only inhibit Aβ aggregation but also photodegrade Aβ aggregates, such as Aβ oligomers. Meanwhile, Li *et al.*^254^ designed bifunctional nanoparticles POM@P through the self-assembly of Aβ_15–20_ peptides and POM (Fig. 15A). The aggregation process of Aβ was researched by monitoring the fluorescence of Congo red's after adding POM@P. Moreover, the prepared POM@P could effectively target amyloid aggregation in mouse cerebrospinal fluid. The interaction between POMs and Aβ species relies on an electrostatic effect. Gao *et al.*^255^ designed a variety of transition-metal-substituted POMds that had better inhibition efficiency of Aβ aggregation than POMs. Results demonstrated that POMds with histidine-binding sites could not only specifically target the polypeptide sequence (HHQK) of Aβ, but also show stronger inhibitory effects through enhancing binding affinity between Aβ and POMds (Fig. 15B). POMds-Dawson-Ni and POMds-Dawson-Co exhibited better effects for decreasing Aβ-haem peroxidase-like activity. In addition, POMds could across the BBB and were metabolized completely after 48 hours. Then Gao continuously designed artificial enzymes AuNPs@POMD-8pep that exhibited protease activities, SOD-like functionality, and metal-ion chelation capabilities (Fig. 15C).^256^ AuNPs facilitated electron transfer and served as a scaffold to create a coupled POMD-peptide compound.

Aβ fibrils and ROS are closely related to AD pathogenesis. The reduced POMs (rPOMs) had a strong NIR absorption and ability for anti-oxidant activity.^257–259^ Ma *et al.* designed a NIR-responsive rPOM-based agent rPOMs@MSNs@copolymer that consists of mesoporous silica nanoparticles (MSNs), rPOMs, and thermal responsive copolymer poly(*N*-isopropylacrylamide-*co*-acrylamide).^252^ The copolymer could melt under 808 nm irradiation and led to the release of rPOMs. Therefore, preformed Aβ fibrils could be disaggregated by local heat. Zhao *et al.* reported an organic platinum-substituted POM with a Kegging structure (Me_4_N)_3_[PW_11_O_40_(SiC_3_H_6_NH_2_)_2_PtCl_2_] (abbreviated as Pt^II^-PW_11_) (Fig. 15D).^29^ The negatively charged Pt^II^-PW_11_ anions could bind to the cationic cluster (HHQK) of Aβ through electrostatic interaction, and Pt^II^-PW_11_ interacted with other residues through van der Waals force, hydrogen bonding and desolvation energy. Pt^II^-PW_11_ also reduced Aβ_42_ aggregation-induced cytotoxicity. When the dosage reached 8 μM, cell viability increased from 49% to 67%. In 2022, Gao innovatively combined post-translational modification (PTM) technology with POMs, rationally designed and synthesized a Wells–Dawson POM-based PTM agent POMD-TZ (thiazolidinethione as TZ) for chemical modification of amyloid peptides.^260^ POMD-TZ could selectively bind to the Lys16 site, inhibit Aβ aggregation, and reduce the cytotoxicity caused by the Aβ peptide.

### 5.3 Liposomes

Liposomes have the advantages of non-toxicity, strong drug-carrying capacity, and ease of synthesis and modification, and have been widely used in the field of drug delivery.^261,262^

Gobbi *et al.*^263^ reported that nanoliposomes containing phosphatidic acid (PA) and cardiolipin (CL) targeted aggregated forms of Aβ_42_ fibrils (22–60 nM) with high binding affinity. Mourtas *et al.*^240^ successfully used click chemistry to decorate the surface of nanoliposomes with curcumin, and the curcumin-modified liposomes (maintaining the planarity) had extremely high affinity for Aβ_42_ fibers (1–5 nM) and had sufficient stability for *in vivo* applications. This high-affinity binding may be due to a multivalent interaction between click curcumin liposomes and Aβ. Taylor *et al.*^264^ designed and formulated different types of nanosized liposomes incorporating or decorated with curcumin, a curcumin derivative, or lipid ligands (PA, CL, or GM1 ganglioside), and then evaluated their ability to influence Aβ_42_ peptide aggregation based on ThT and a sandwich immunoassay. The results showed that the click-curcumin type was by far the most effective. Bana *et al.*^265^ prepared phosphatidic acid and ApoE-derived peptide bi-functionalized mApoE–PA–LIP. mApoE–PA–LIP strongly bound the Aβ peptide (kD = 0.6 μM), inhibited peptide aggregation and triggered preformed aggregates. The permeation rate across the BBB of mApoE–PA–LIP was 5-fold higher with respect to mono-functional liposomes. Papadia *et al.*^266^ developed multifunctional LUV liposomes (mf-LIPs) having three ligands, one of which is a curcumin-lipid ligand (TREG) and the other two ligands target the transferrin and the LDL receptors of the BBB. Further research found that the multiple ligands of mf-LIPs did not interfere with each other, and mf-LIPs have multiple functions such as targeting the BBB and inhibiting amyloid aggregation. Meanwhile, *in vivo* experiments found that the curcumin ligand increases the stealth properties of liposomes by reducing their uptake by the liver and spleen.^267^ Kuo *et al.*^268^ designed a drug carrier system of ApoE-modified liposomes conjugated with PA. This system was used to improve BBB penetration and release quercetin (QU) and rosmarinic acid (RA) to inhibit Aβ_42_. ApoE–QU–RA–PA–liposomes could penetrate the BBB because of strong attraction between low-density lipoprotein receptors and ApoE.

### 5.4 SiO_2_

Silica nanostructures, due to their synthetic flexibility, molecular properties, multifunctionality, and biocompatibility, have long been used in biomedical applications.^269,270^

In 2016, Hulsemann *et al.*^241^ reported a highly stable standard in the size range of native Aβ oligomers consisted of a silica nanoparticle, which is functionalized with Aβ peptides on its surface (Aβ-SiNaP). The detection limit corresponded to an Aβ concentration of 1.9 ng L^−1^. Zhang *et al.*^271^ synthesized β-NaYF_4_:Yb/Er@SiO_2_@RB by combining upconversion nanoparticles (UCNPs) with photosensitizers to disaggregate the preformed Aβ aggregates under NIR light. UCNPs were able to transfer energy to RB at 980 nm and Aβ_42_ fibrils were disaggregated *via* photo-induced ROS. Jung *et al.*^30^ designed Aβ nanodepletors consisting of ultralarge mesoporous silica nanostructures and anti-Aβ single-chain variable fragments (anti-Aβ scFvs). The Aβ nanodepletors suppressed Aβ self-assembly, decreased the amount of Aβ aggregates, and increased cell viability.

In addition, many other nanomaterials have been reported for AD diagnosis and treatment, such as carbon nanospheres, hydrogen-bonded organic frameworks (HOFs), polystyrene nanoparticles and so on. Ma *et al.*^272^ designed NIR-II photothermally responsive mesoporous carbon nanospheres KD8@N-MCNs. The graphitic N dopants introduced abundant electrons into the p* orbital between the HOMO and LUMO gaps, thus enhancing the light absorption properties. Under 1064 nm light irradiation, the nanospheres disaggregated Aβ_42_ aggregates because of photothermal conversion ability. Meanwhile, KD8@N-MCNs alleviated oxidative stress due to the SOD and CAT enzymatic activities. Due to the covalently grafted KLVFFAED, KD8@N-MCNs could cross the BBB and specifically recognize Aβ_42_ aggregates. HOF materials exhibit considerable biocompatibility and low toxicity attributed to their metal-free nature, thus being an excellent candidate for drug delivery and biological applications.^273^ Zhang *et al.* designed a two-photon NIR-II-activated photooxygenation catalyst DSM@*n*-HOF-6 (DSM = 4-[*p*-(dimethylamino) styryl]-1-methylpyridinium). TCPP(*meso*-tetrakis(carboxy phenyl)porphyrin) was periodically incorporated into HOFs, while the targeting peptide KLVFFAED (KD8) was conjugated to DSM@*n*-HOF-6 (DSM@*n*-HOF-6@KD8).^274^ The up-conversion fluorescence of DSM could be absorbed by TCPP to generate ^1^O_2_ for Aβ oxygenation, and DSM@*n*-HOF-6@KD8 could inhibit the fibrillation of Aβ monomers and reduce the cytotoxicity of Aβ by photooxygenation. The application of polystyrene nanoparticles and upconverting nanoparticles in amyloid diseases has also been reported.^275,276^

Many nanomaterials have been reported for AD diagnosis and treatment. Targeting issues and the interaction between nanomaterials and peptides are the main issues that need to be considered. At the same time, issues such as inhibition efficiency, reversibility of fibrosis, and nanoparticle metabolism also need to be considered.

## 6 Conclusions and outlook

Due to the intensification of the aging of the population, neurodegenerative diseases, especially AD, have become one of the most serious obstacles to social development. This review comprehensively summarized the recent research for modulating amyloid aggregation associated with neurodegenerative diseases, including AD based on nanomaterials and nanotechnology. In this review, nanomaterials exhibited multiple roles in the treatment of AD. Firstly, nanomaterials can directly interact with Aβ peptides and accelerate or slow down amyloid aggregation. Secondly, as nanocarriers, nanomaterials can also be used to load various drugs and assist drugs to across the BBB and inhibit amyloid. In addition, nanomaterials and drugs can synergistically resist a series of problems arising from amyloid aggregation. Moreover, some advanced nanomaterials with photosensitivity can strongly affect amyloid aggregation through PTT or PDT.^277^ Multiple interaction mechanisms such as electrostatic interaction, hydrophobic interaction, π–π stacking, and metal ion chelation are the main reasons for amyloid fibrillation, and taking advantage of these interactions in the design process and application process, nanomaterials can effectively adsorb amyloids on their surface and block the amyloid aggregation. As novel treatment methods, advanced nanomaterials with the function of PTT and PDT have the advantages of accuracy, ease of administration, high efficiency, and few side effects. In PDT treatment, nanomaterials can generate ROS and oxidize the amino acid residue of amyloid, and then the aggregation process of amyloid is inhibited. In PTT treatment, because the amyloid formation is highly dependent on temperature, the change of localized temperature can affect amyloid aggregation. Currently, PDT and PTT have attracted more and more attention from researchers, and these methods may be the focus of follow-up research. In the future, a deep and comprehensive understanding of the functional design of nanomaterials and the properties of these nanomaterials is still necessary. The mechanism of action of nanomaterials in AD treatment, especially the in-depth research mechanism of amyloid, including interaction and photo-inhibition process, also needs to be further explored. From the current point of view, the development of nanomaterials has shown a new chapter in the treatment of AD.

Regarding the application of nanomaterials for inhibiting amyloid, there are several aspects to note: (1) in order to successfully achieve clinical applications, it is necessary to further understand and explore the *in vivo* distribution and metabolism of nanomaterials. In future research, it is necessary to continue in-depth research on the biological properties, preparation processes, and surface modification of nanomaterials to achieve a more safe and more efficient inhibition of amyloid aggregation and disaggregation of amyloid fibrils. (2) The BBB permeability of nanomaterials can be improved through some conjugates such as transferrin (*via* transferrin receptor-mediated endocytosis to cross the BBB), and transiently disrupting the blood–brain barrier by physical methods such as photothermal and intranasal administration may also be an effective method. (3) To predict the interactions between amyloid/fibrils and nanoparticles in advance, it is also necessary to build suitable computational models and deeply explore how amyloid/fibrils and nanoparticles interact. (4) Most nanomaterials interact with amyloid through non-covalent interactions, which are weak and may cause reversible aggregation/disaggregation processes, and covalent modulators can prolong their duration of action. PDT and PTT also can directly irreversibly modulate the amyloid fibrosis process. (5) The role of nanomaterials and amyloid at the cellular level and *in vivo* is worth further research in the future, which will provide a strong basis for a biological experiment for nanomaterials to transform nanomedicines. (6) For precise inhibition, the problem of targeting needs to be solved urgently. Targeting ligands have been introduced, such as antibodies, peptides, and aptamers. The amyloid targeting ligand may lose or weaken its binding affinity to amyloid during PDT or PTT, so it is crucial to develop targeting ligands with high stability and strong affinity under harsh conditions. (7) Many studies have pointed to oligomers, and the subsequent application of nanomaterials in oligomers will become the focus and be further explored. (8) Although studies have shown that nanomaterials have a good inhibition efficiency *in vitro*, the inhibition efficiency needs to be further improved *in vivo*. To prove whether the addition of nanomaterials can restore the normal function of nerve cells, various experiments *in vivo* including clinical research still need to be carried out.

## Conflicts of interest

The authors declare no conflict of interest.

## Supplementary Material
